# Deoxycholate induces proline provision from *Escherichia coli* to enhance *Clostridioides difficile* virulence

**DOI:** 10.1093/ismejo/wrag249

**Published:** 2026-09-17

**Authors:** Jingpeng Yang, Xilong Deng, Xiaoya Sheng, Yanan Li, Xuna Tang

**Affiliations:** Affiliated Hospital of Medical School, Nanjing Stomatological Hospital, Nanjing University Joint Research Center of Nanjing Normal University and Nanjing Stomatological Hospital, Nanjing, Jiangsu 210008 and 210033, China; State Key Laboratory of Microbial Technology, Nanjing Normal University, 2 Xuelin Road, Qixia District, Nanjing, Jiangsu 210033, China; Affiliated Hospital of Medical School, Nanjing Stomatological Hospital, Nanjing University Joint Research Center of Nanjing Normal University and Nanjing Stomatological Hospital, Nanjing, Jiangsu 210008 and 210033, China; State Key Laboratory of Microbial Technology, Nanjing Normal University, 2 Xuelin Road, Qixia District, Nanjing, Jiangsu 210033, China; Affiliated Hospital of Medical School, Nanjing Stomatological Hospital, Nanjing University Joint Research Center of Nanjing Normal University and Nanjing Stomatological Hospital, Nanjing, Jiangsu 210008 and 210033, China; State Key Laboratory of Microbial Technology, Nanjing Normal University, 2 Xuelin Road, Qixia District, Nanjing, Jiangsu 210033, China; Affiliated Hospital of Medical School, Nanjing Stomatological Hospital, Nanjing University Joint Research Center of Nanjing Normal University and Nanjing Stomatological Hospital, Nanjing, Jiangsu 210008 and 210033, China; State Key Laboratory of Microbial Technology, Nanjing Normal University, 2 Xuelin Road, Qixia District, Nanjing, Jiangsu 210033, China; Affiliated Hospital of Medical School, Nanjing Stomatological Hospital, Nanjing University Joint Research Center of Nanjing Normal University and Nanjing Stomatological Hospital, Nanjing, Jiangsu 210008 and 210033, China; Affiliated Hospital of Medical School, Nanjing Stomatological Hospital, Nanjing University, 30 Central Road, Xuanwu District, Nanjing, Jiangsu 210008, China

**Keywords:** *Clostridioides difficile* infection, deoxycholate, *Escherichia coli*, L-proline, D-proline reductase

## Abstract

*Clostridioides difficile* infection (CDI) is a refractory enteritis. A key indicator of successful treatment and microbial restoration is the elevation of intestinal deoxycholate (DCA) levels. Nevertheless, a small proportion of patients experience disease recurrence despite this apparent metabolic recovery, the underlying mechanisms of which remain poorly understood. We investigated the ecological dynamics of *Clostridioides difficile* (CD) and *Escherichia coli* (EC) under DCA exposure, revealing that DCA exacerbates the severity of co-infection. Specifically, DCA promotes the formation of a robust and dense symbiotic biofilm that confers a substantial survival advantage to CD. Through a combination of *in vitro* co-culture and validation in a murine infection model, we demonstrated that EC produces substantial quantities of L-proline in response to DCA. This amino acid is converted to D-proline by the proline racemase and subsequently catabolized by the D-proline reductase in CD, thereby enhancing its environmental fitness and pathogenicity within the gut. The indispensable nature of this tripartite interaction was further corroborated using additional EC or CD strains, including isogenic mutants deficient in D-proline reductase, in subsequent murine infection models. These experiments collectively confirm that EC, proline, and the D-proline reductase are all essential components mediating the DCA-induced exacerbation of CDI. In summary, our study elucidates a mechanism by which a DCA environment promotes the exacerbation of CDI through a metabolic cross-feeding interaction between CD and EC. Specifically, EC-derived L-proline serves as a critical nutrient that enhances the fitness and pathogenicity of CD. This finding provides a plausible explanation for the paradoxical phenomenon of CDI recurrence in patients exhibiting elevated intestinal DCA levels.

## Graphical Abstract

**Graphical Abstract:**
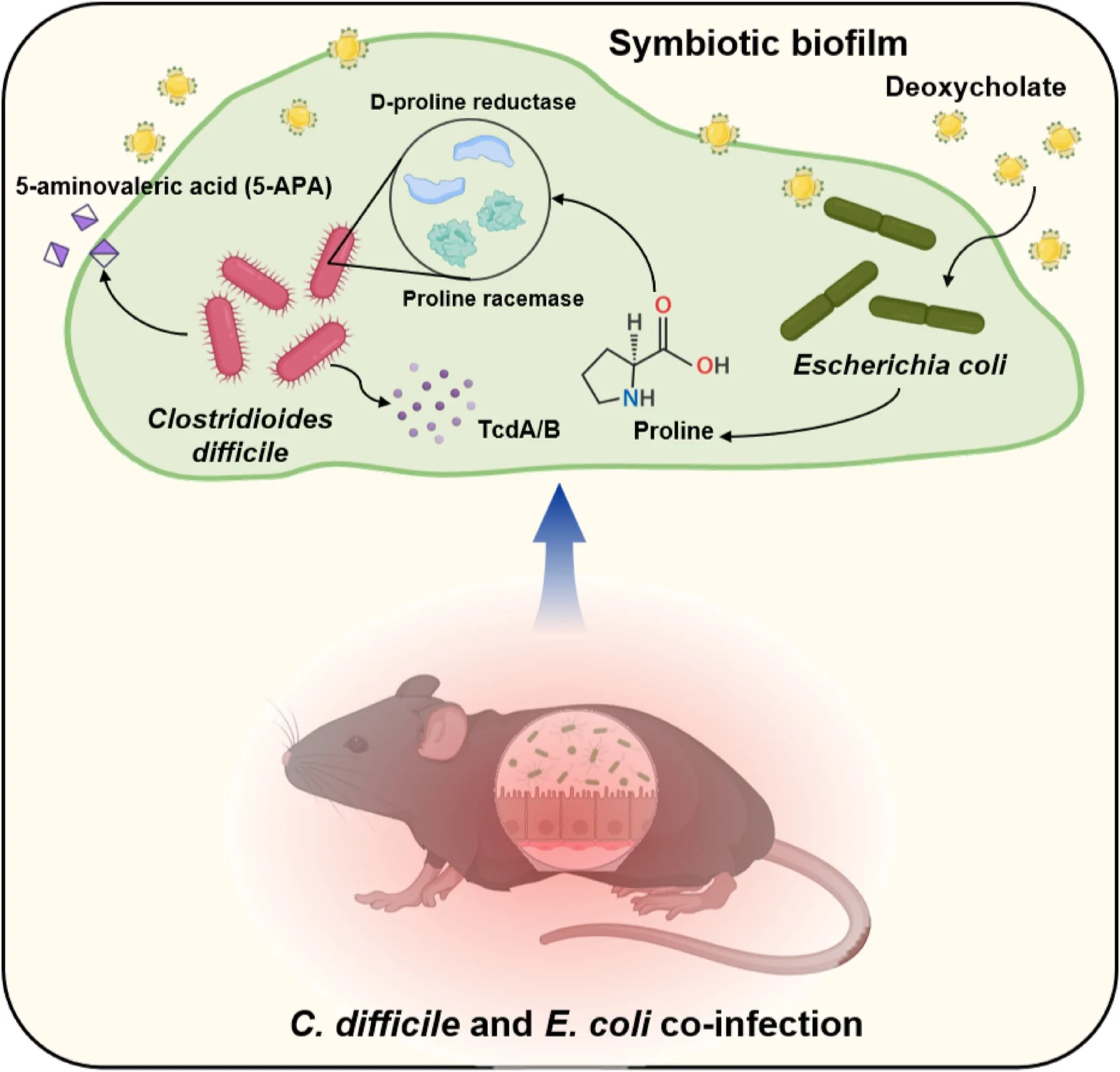

## Introduction

*Clostridioides difficile* infection (CDI) imposes a substantial and growing economic burden on healthcare systems worldwide. The current standard of care for primary CDI (pCDI) predominantly involves antibiotic therapy, with agents such as vancomycin (VAN) and fidaxomicin (FID) being the mainstays of treatment. However, for persistent or recurrent CDI (rCDI), which represents a clinical challenge, fecal microbiota transplantation (FMT) has emerged as a highly effective therapeutic modality [1]. Both antibiotic therapy and FMT, whereas employed for the prevention and treatment of pCDI or rCDI, profoundly perturb the host’s intestinal ecosystem. These interventions induce shifts in microbial composition and the metabolic landscape, including the concentrations of key metabolites. Consequently, these ecological alterations critically influence the trajectory of the infection and the efficacy of treatment through both direct and indirect approaches [2–4].

Bile acids (BAs), a critical class of metabolites, are broadly categorized into primary and secondary forms [5]. Primary bile acids (PBAs), such as cholic acid (CA), act as potent germinants for *C. difficile* (CD) spores, thereby facilitating the initiation of infection. In contrast, secondary bile acids (SBAs), such as deoxycholic acid (deoxycholate, DCA), are known to inhibit both vegetative cell growth and spore germination [6]. Consequently, SBAs, particularly DCA, are considered pivotal for host defense and for maintaining intestinal homeostasis [7–9], with elevated intestinal DCA levels being widely regarded as a hallmark of a healthy, recovered gut microbiome [6, 10]. Nevertheless, a notable clinical paradox persists: some patients experience disease recurrence even after their intestinal DCA levels have restored to levels comparable to those of healthy individuals [11, 12]. The mechanisms underlying this refractory recurrence remain poorly understood. Emerging evidence highlights the role of intestinal DCA in promoting bacterial biofilm formation. Aguirre *et al*. [13] demonstrated that DCA induces CD biofilm formation at specific concentrations, whereas Dubois *et al*. [14] further elucidated that DCA functions as an intestinal signaling molecule to trigger this process, ultimately facilitating bacterial persistence. These findings offer a plausible explanation for the aforementioned clinical paradox, suggesting that the restoration of DCA represents only one component of a multifactorial protective response and is insufficient in isolation to prevent recurrent rCDI.

Mounting evidence indicates that certain gut microbes can engage in synergistic relationships with CD, enhancing its resilience to antimicrobial agents and BAs [15]. A prominent example is the family *Enterococcaceae*, whose expansion is strongly correlated with CDI severity. For instance, *Enterococcus faecalis* capitalizes on host hemoglobin released from CD toxin-induced tissue damage, thereby boosting its own fitness [16]. Concurrently, *E. faecalis* reciprocally fuels CD growth by supplying fermentable amino acids like leucine and ornithine, which exacerbates the infection [17]. Our previous research further reveals a distinct protective mechanism, wherein *Bacteroides thetaiotaomicron* forms a robust biofilm with CD. This symbiotic biofilm, induced by VAN exposure, confers substantial VAN resistance upon CD [15]. Collectively, these intricate cross-species interactions underscore how specific members of the gut microbiota can facilitate CD persistence, contributing to the establishment of chronic and refractory infections.

The *Enterobacteriaceae* family represents another group of microbes whose abundance is consistently elevated in cases of refractory or worsening CDI, though their precise role in this context remains to be elucidated [18, 19]. A considerable clue lies in the well-established link between *Enterobacteriaceae* expansion and inflammatory bowel disease (IBD), a condition that increases the risk of CDI by approximately fourfold [20]. This suggests that an inflammatory gut environment may foster a niche where *Enterobacteriaceae* can promote CD pathogenicity. Indeed, *Enterobacteriaceae* are not merely passive inhabitants; they are known to be key modulators of intestinal homeostasis, immune responses, and even drug efficacy. A comprehensive metagenomic analysis of over 12 000 human gut microbiomes confirmed that members of this family, such as *Escherichia coli* (EC) and *Klebsiella pneumoniae*, are major contributors to opportunistic infections [21]. This study also demonstrated that the over-proliferation of *Enterobacteriaceae*, irrespective of pathogenicity, is broadly associated with increased infection risk, non-communicable diseases like IBD, and higher all-cause mortality [21]. Providing a direct mechanistic link, a recent study showed that BAs-induced alterations in the gut metabolome can enhance the colonization capacity of *Enterobacteriaceae*, particularly EC [22]. Collectively, these lines of evidence position *Enterobacteriaceae* as a potential accomplice in the exacerbation of CDI.

We hypothesized that in a DCA environment, CD establishes a collaborative relationship with EC, leading to exacerbated infection. To test this, we first investigated the ecological interactions between CD and EC under DCA conditions *in vitro*, focusing on biofilm formation and the transcriptional and metabolic responses of CD. Our initial findings revealed that DCA induces the formation of a robust and dense symbiotic biofilm and concurrently upregulates the expression level of D-proline reductase in CD in a dose-dependent manner. Subsequently, using a murine CDI model, we validated these interactions *in vivo* and, through metabolomics, identified D-proline as a key metabolite enriched in this context. Further analysis confirmed that EC supplies substantial quantities of L-proline in a DCA environment, which in turn promotes CD growth and toxin production. Finally, the universality of this DCA-mediated exacerbation was confirmed using various CD and EC strains in additional mouse models. Collectively, these results identify EC-derived L-proline, which is converted to D-proline by the proline racemase and subsequently catabolized by the D-proline reductase in CD, and the presence of EC as essential factors driving the pathogenesis of CDI under DCA conditions.

## Materials and methods

### Cultivation of strains

*Clostridioides difficile* ATCC 43255 (CD), *C. difficile* ATCC 9689, *E. coli* ATCC 25922 (EC), and *E. coli* K-12 (ATCC 25404) were purchased from the American Type Culture Collection (ATCC; Manassas, VA, USA). *C. difficile* CS11, *C. difficile* CS25, and *E. coli* CS05 are clinical isolates preserved in our laboratory. *C. difficile* 630 and *C. difficile* 630 (*ΔprdB*) were provided by Professor Hong Yang from Shanghai Jiao Tong University. *C. difficile* is inoculated into Brain Heart Infusion (BHI) broth or Lysogeny Broth (LB) broth, or spread onto Taurocholate Cycloserine Cefoxitin Fructose Agar (TCCFA, BioWORLD, USA) for cultivation. *E. coli* is inoculated into BHI or LB broth, or spread onto *E. coli* Chromogenic Medium (ECCM, Hope Bio, China) for cultivation. Both were cultured in an anaerobic workstation (85% nitrogen, 10% hydrogen, 5% carbon dioxide; ELECTROTEK AW 400TG TWO GAS VERSION) maintained at 37°C. Sodium deoxycholate (30970-100G, DCA) was purchased from Sigma-Aldrich (Merck, Germany).

### Ecological interplay between *C. Difficile* and *E. Coli* in DCA-depleted environments

Cell suspensions and cell-free culture supernatants (CFCS) of CD and EC were prepared for cross-treatment experiments. The effects of these reciprocal CFCS on the growth and biofilm formation of both bacteria were evaluated by measuring optical density and using crystal violet staining. Additionally, toxin A and B levels in CD exposed to EC-CFCS were quantified using a commercial ELISA kit (Detailed methods are in the supplementary materials).

### Impact of DCA on the growth and metabolism of *C. Difficile* and *E. Coli*

To determine the effect of DCA on bacterial proliferation, a two-fold serial dilution of DCA was prepared in fresh Brain-Heart Infusion (BHI) broth. Aliquots of 180 μL from each dilution were dispensed into the wells of a 96-well microplate. Subsequently, 20 μL of either CD or EC cell suspension was added to the corresponding wells. The plate was then incubated anaerobically at 37°C for 24 h. Bacterial growth was quantified by measuring the optical density at 630 nm using a microplate reader. Wells containing BHI broth without DCA served as the growth control. For the analysis of DCA metabolism, CD and EC cultures were first grown to the late exponential phase and standardized to a similar optical density. These cultures were then inoculated into fresh BHI broth supplemented with a specific concentration of DCA (1 mM). Following anaerobic incubation at 37°C, 1 mL samples were collected at the initial time point (0 h) and after 24 h. The samples were centrifuged to pellet the cells, and the resulting supernatants were collected. The concentration of residual DCA in the supernatants was quantified using a commercial DCA assay kit (MEIAO BIOTECH, Shanghai, China) according to the manufacturer’s instructions.

### DCA-mediated interplay between *C. Difficile* and *E. Coli*

To establish monocultures, 50 μL aliquots of either CD or EC cell suspensions, normalized to an equivalent optical density at 630 nm, were inoculated into 150 μL of fresh BHI broth. For co-culture experiments, 50 μL of CD-cell suspension was combined with 50 μL of EC-cell suspension, and the mixture was then inoculated into 150 μL of fresh BHI broth. For both experimental setups, parallel controls were established using an equal volume of BHI broth without DCA. All microplates were incubated anaerobically at 37°C. At the 24 h time point, biomass and CD toxin content were assessed. Biofilm formation was subsequently quantified at 48 hours.

### DCA as a modulator of the *C. Difficile*-*E. Coli* interplay: Consequences for growth, biofilm, and toxin production

A two-fold serial dilution was performed from a 1 mM DCA stock solution to generate a concentration gradient (1, 0.5, 0.25, 0.125, 0.0625, and 0.03125 mM). Fresh BHI broth without DCA served as the negative control (0 mM). Co-cultures were prepared by combining 50 μL of CD cell suspension with 50 μL of EC cell suspension. A 100 μL aliquot of this bacterial mixture was then inoculated into each well of a 96-well plate containing 100 μL of the prepared DCA solutions at the specified concentrations. Following an appropriate incubation period, the cultures were assessed for biomass, biofilm formation, and CD toxin production.

### Dose-dependent chemotaxis and aggregation of *C. Difficile* in response to DCA

DCA-pretreated CD and EC cells were used for chemotaxis and aggregation assays. The Chemotactic Index (CI) was determined by the fluorescence of labeled CD cells migrating toward chemoattractants in capillaries, while the Aggregation Index (AI) was calculated from the OD600 changes during a 1-hour co-incubation of CD and EC cells (detailed methods are in the supplementary materials).

### Quantification of biofilm thickness and viable cell density via FISH-CLSM

Based on preliminary DCA gradient experiments, three concentrations were selected for subsequent analysis: low (0.03125 mM; CEL), medium (0.125 mM; CEM), and high (1 mM; CEH). Biofilms were formed on sterile round coverslips placed at the bottom of 12-well plates. CD and EC cultures were adjusted to a final concentration of 3 × 10^7^ CFU/mL. For monocultures, 100 μL of a single strain was added to 2900 μL of BHI broth. For co-cultures, 100 μL of CD and 100 μL of EC were added to 2800 μL of BHI medium, either with or without the specified DCA concentrations. All plates were incubated anaerobically at 37°C for 48 hours to facilitate biofilm development. To visualize the spatial distribution of each microbe within the biofilm, FISH was performed (Detailed methods are in the supplementary materials).

### Modulation of *C. Difficile* morphology and motility by DCA

To assess dose-dependent effects, CD was treated with low, medium, or high DCA concentrations in monoculture or co-culture with EC using transwell chambers, and morphological changes were analyzed via Gram staining after 48 hours. Additionally, the swimming and swarming motility of the treated CD cells were evaluated on 0.3% agar plates by measuring the growth diameters daily for 5 days (Detailed methods are in the supplementary materials).

### RNA-sequencing (RNA-seq) analysis

CD and EC were co-cultured using a transwell system and treated with varying concentrations of DCA for 16 hours. Following incubation, CD cells were harvested for transcriptome sequencing, while cell-free culture supernatants were collected for metabolomics analysis (detailed methods are in the supplementary materials).

### Untargeted or targeted metabolomic profiling

Metabolites were extracted from fresh samples (bacterial suspensions or mouse feces). An aliquot of each sample was mixed with an ice-cold solvent mixture (methanol: acetonitrile: water, 2:2:1, v/v) and vortexed thoroughly. The mixture was then sonicated for 30 min at −20°C, followed by a 10-min incubation on ice. After centrifugation at 14 000 × g for 20 min at 4°C, the resulting supernatant was collected and dried under vacuum. The dried residue was reconstituted in 100 μL of a 1:1 (v/v) acetonitrile-water solution, vortexed, and centrifuged. The final supernatant was transferred to autosampler vials for LC–MS analysis (Detailed methods are in the supplementary materials).

### Murine model of CDI

**Experiment 1:** Six-week-old male C57BL/6 J mice were purchased from Shanghai Slake Experimental Animal Co., Ltd. (Shanghai, China) and acclimatized for one week before experimentation. All mice were housed individually under identical conditions and confirmed to be CD toxin-negative before the study. All animal procedures were approved by the Animal Ethics Committee of Nanjing Normal University (IACUC-2603044). A CDI model was established by adapting previously reported protocols [17, 23, 24]. Mice were randomly assigned (*n* = 10 per group) to one of eight groups: two control groups (NCn and NCa), two single-bacterium infection groups (CD and EC), one co-infection group (CE), and three co-infection groups with DCA treatment (CEL, CEM, CEH). Except for the NCn group, all mice received a 7-day course of antibiotics (1 mg/mL cefoperazone) in their drinking water to disrupt the gut microbiota, followed by a 2-day recovery period on normal water. Subsequently, the infection phase was initiated via oral gavage. Mice in the CD group were inoculated with 3 × 10^8^ CFU of *C. difficile* ATCC 43255. Mice in the EC group received 3 × 10^8^ CFU of *E. coli* ATCC 25922. Mice in the co-infection groups (CE, CEL, CEM, CEH) were inoculated with a suspension containing both 3 × 10^8^ CFU of *C. difficile* and 3 × 10^8^ CFU of *E. coli*. For the DCA treatment groups (CEL, CEM, and CEH), mice were subjected to oral gavage with escalating concentrations of DCA (0.03125 mM, 0.125 mM, and 1 mM, respectively) beginning 12 hours post-infection, and maintained for the duration of the study (until CDI symptoms disappeared). Throughout the experiment, mice were monitored daily for body weight, survival, and clinical signs of disease. Disease severity was scored on a scale from 0 (normal) to 20 (near death) [25]. Humane endpoints were strictly observed; mice were euthanized when they became moribund or exhibited weight loss exceeding 20% of their baseline body weight. Fecal samples were collected at four key time points: T1 (pre-antibiotic), T2 (pre-infection), T3 (one day post-infection), and T4 (study endpoint). These samples were used for quantification of viable *C. difficile* and *E. coli*, measurement of CD toxin and fecal lipocalin-2 (Lcn2), and for microbiome and metabolome analyses. Detailed methods of experiments 2, 3, 4, 5, and 6 are in the supplementary materials.

### Determination of the levels of some key bile acid production enzymes in liver

Hepatic tissues were harvested from mice following seven consecutive days of intragastric DCA administration. With slight modifications based on the method described by Chen *et al*. [26], mouse livers were harvested, weighed, and immediately snap-frozen in liquid nitrogen for subsequent analysis (detailed methods are in the supplementary materials).

### Histopathological evaluation and clinical disease assessment

Cecal and colonic tissues were harvested and fixed in 10% neutral-buffered formalin. After paraffin embedding, 5 μm sections were prepared and stained with Hematoxylin and Eosin (H&E). Histopathological scoring was performed by a blinded observer according to a previously established system [23] (detailed methods are in the supplementary materials).

### Quantification of fecal *C. Difficile* and *E. Coli* loads and toxin levels

Freshly collected fecal samples were weighed, homogenized in sterile phosphate-buffered saline (PBS), and subjected to serial tenfold dilutions. For the enumeration of CD, aliquots of the dilutions were spread-plated onto TCCFA and incubated under anaerobic conditions. EC was quantified by plating onto *E. coli* Chromogenic Medium (ECCM). Bacterial loads were expressed as colony-forming units per gram of feces (CFU/g). Concurrently, the concentrations of CD toxins A and B (TcdA/TcdB) in the fecal supernatants were determined using a *C. difficile* TOXA/B II™ kit (TechLab, Blacksburg, VA, USA), following the manufacturer’s instructions.

### Measurement of fecal lipocalin-2 (Lcn-2) levels

Frozen fecal samples were weighed and homogenized in phosphate-buffered saline (PBS) containing 0.1% Tween 20 at a concentration of 100 mg/mL. The suspensions were vortexed for 20 min to ensure uniformity and then centrifuged at 14 000 × g for 10 min at 4°C. The resulting supernatants were collected and stored at −20°C until further analysis. The concentration of lipocalin-2 (Lcn-2) in the fecal supernatants was quantified using the Duoset Mouse Lcn-2 ELISA Kit (R&D Systems, Minneapolis, MN, USA), strictly following the manufacturer’s instructions. The assay was developed with tetramethylbenzidine (TMB) substrate, and the optical density (OD) was measured at a wavelength of 450 nm [27].

### Fecal microbiota profiling by 16S rRNA gene sequencing and shotgun metagenomics

Fecal genomic DNA was extracted using the OMEGA Soil DNA Kit (D5625–01, Omega Bio-Tek, Norcross, GA, USA) according to the manufacturer’s protocol. The concentration and purity of DNA were measured using a NanoDrop ND-1000 spectrophotometer (Thermo Fisher Scientific, Waltham, MA, USA), whereas its integrity was verified by 1.2% agarose gel electrophoresis. Purified DNA was stored at −20°C until further processing (detailed methods are in the supplementary materials).

### Effects of specific metabolites on microbial growth in the presence of DCA

An integrative analysis of previous *in vitro* metabolomics data with murine fecal microbiome and metabolome profiles identified a panel of metabolites significantly associated with the abundance of *C. difficile* 43 255 and *E. coli* 25 922. These metabolites included L-arginine, 5-aminovaleric acid (5-APA), N-acetylornithine, L-isoleucine, D-proline, and D-ornithine. To functionally validate their impact on bacterial proliferation, we conducted growth assays. *C. difficile* 43 255 or *E. coli* 25 922 were inoculated into fresh BHI broth, either unsupplemented (control) or individually supplemented with 1 mM of each respective metabolite. The cultures were then incubated at 37°C under anaerobic conditions, and bacterial growth was monitored over time.

### Impact of DCA on D-proline metabolism, gene expression, and toxin production in *C. Difficile*

To investigate the metabolic interaction between *C. difficile* and *E. coli*, we inoculated *C. difficile* 43 255 and *E. coli* 25 922 (3 × 10^7^ CFU/mL each) into fresh BHI broth containing 1 mM D-proline, in the presence or absence of 1 mM DCA. Cultures were incubated for 24 hours in mono- and co-culture formats. Subsequently, D-proline consumption was quantified, and the concentrations of 5-APA and CD toxin proteins (TcdA and TcdB) were measured. In parallel, *E. coli* 25 922 was cultured in fresh BHI broth lacking exogenous proline, with or without 1 mM DCA supplementation, to quantify the concentrations of D- and L-proline in the culture supernatant. To assess the broader applicability of these findings, the study was replicated using additional *C. difficile* strains (9689, CS25, 630) and an *E. coli* strain (CS05). Furthermore, quantitative real-time PCR (qPCR) analysis was performed to evaluate gene expression. Using the 2^-ΔΔCt^ method and strain-specific primers (Table S1), we determined the transcriptional levels of *prdF, prdB, tcdA*, and *tcdB* in *C. difficile* 43 255, and *proB* and *putP* in *E. coli* 25 922 [15].

### Bioinformatics analysis

Integrate transcriptomic, metabolomic, and microbiome data to conduct a comprehensive multi-omics analysis (detailed methods are in the supplementary materials).

### Statistical analyses

For all other statistical comparisons between two or more groups, GraphPad Prism 10 (GraphPad Software, LLC, USA) was utilized. A *P*-value of < .05 was considered statistically significant.

## Results

### DCA induces the formation of a dense symbiotic biofilm between *C. Difficile* and *E. Coli*

Initially, we observed that cell-free culture supernatants (CFCS) from CD and EC reciprocally promoted each other’s growth (Fig. 1A and B), but did not significantly alter biofilm biomass when cultured individually (Fig. 1C and D). However, CD produced more toxins in the presence of EC-CFCS compared to monoculture (Fig. 1E). The introduction of DCA fundamentally altered this dynamic. At 1 mM DCA, a concentration inhibitory to CD, EC growth reached its peak (Fig. 1F). Neither organism showed significant utilization of DCA at this concentration (Fig. 1G). Under these conditions, whereas CD’s biomass and toxin production were suppressed, its biofilm formation increased—a trend opposite to that observed in EC. Critically, the presence of DCA in the co-culture enhanced the total biomass, mixed-species biofilm formation, and CD toxin production compared to the DCA-free control (Fig. 1H, I, and  J). This synergistic effect was further confirmed to be dose-dependent. A gradient of DCA concentrations revealed that all measured metrics—biomass, biofilm yield, and toxin levels—were elevated in the presence of DCA, with biofilm formation exhibiting a clear dose–response relationship (Fig. 1K, L, and  M). DCA also enhanced the chemotaxis and auto-aggregation capabilities of CD (Fig. 1N and O). To visualize this, we analyzed biofilms formed at high (1 mM, CEH), medium (0.125 mM, CEM), and low (0.03125 mM, CEL) DCA concentrations. Confocal microscopy confirmed that both biofilm thickness and the viable cell counts of CD and EC within the biofilm increased in a DCA-dependent manner (Fig. 1P, Fig. S1A). Morphologically, CD cells elongated during co-culture but progressively shortened with increasing DCA concentrations (Fig. S1B). Furthermore, motility assays demonstrated that whereas EC co-culture and DCA each independently enhanced CD motility, their combination produced a synergistic amplification of this effect (Fig. 1Q and T). In conclusion, DCA serves as a potent catalyst, mediating highly synergistic interactions between CD and EC. This DCA-induced interaction is characterized by the formation of a robust, mixed-species biofilm, a significant increase in viable bacteria, and the marked enhancement of CD’s pathogenic traits, including toxin production, chemotaxis, aggregation, and motility.

**Figure 1:**
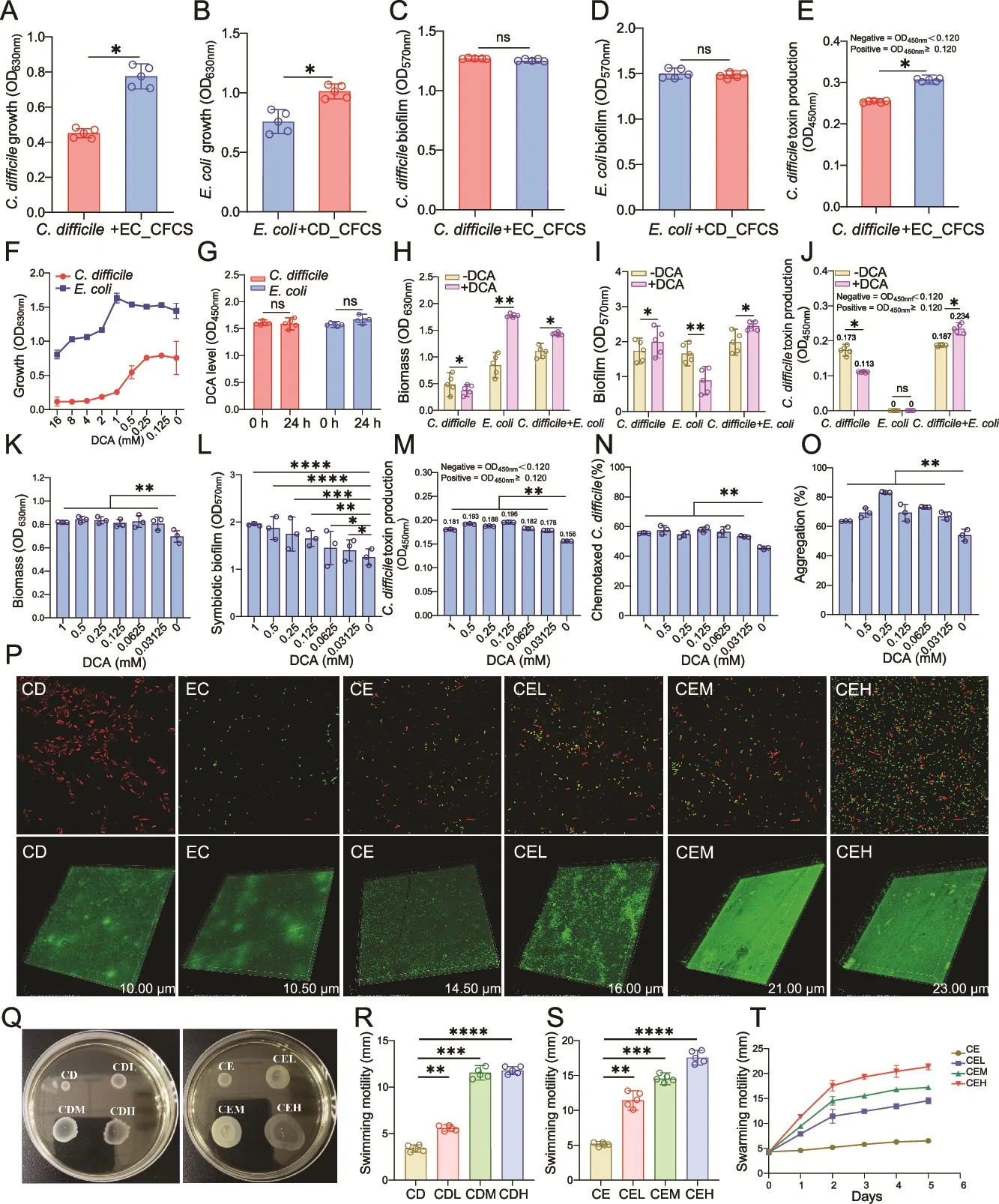
Deoxycholate (DCA) mediates an interaction between *C. Difficile* (CD) and *E. Coli* (EC). A, B. Effects of cell-free culture supernatants (CFCS) from one microbe on the growth of the other. C. Effect of EC-CFCS on CD biofilm yield. D. Effect of CD-CFCS on EC biofilm yield. E. Effect of EC-CFCS on CD toxin production. F. Growth curves of CD and EC in response to a gradient of DCA concentrations. G. Utilization of 1 mM DCA by CD and EC in monoculture. H, I. Biomass and biofilm formation of CD and EC in monoculture versus co-culture with 1 mM DCA. J. CD toxin production in monoculture versus co-culture with 1 mM DCA. K, L, M. Symbiotic biomass, biofilm formation, and CD toxin production under a gradient of DCA concentrations. N. Chemotactic response of CD to a DCA gradient. O. Aggregation capacity of CD and EC in the presence of DCA. P. Representative images of symbiotic biofilms formed at low (CEL, 0.03125 mM), medium (CEM, 0.125 mM), and high (CEH, 1 mM) DCA concentrations. Red fluorescence represents CD, and green fluorescence represents EC. Q, R, S. Swimming motility of CD in the symbiotic system under DCA conditions. T. Swarming motility of CD in the symbiotic system under DCA conditions. For Fig. 1A, B, C, D, E, G, H, I, and  J, statistical significance was determined by a two-tailed Student’s *t-*test; For Fig. 1K, L, M, N, O, R, and  S, statistical significance was determined by Dunnett’s test‌. ns, not significant (*P* > .05); ^*^P < .05; ^**^P < .01; ^***^P < .001; ^****^P < .0001.

### DCA triggered significant transcriptional and metabolic remodeling in co-cultured *C. Difficile*

Using co-cultures (without DCA) as a baseline, we observed a dose-dependent increase in the number of differentially expressed genes (DEGs) in CD with rising DCA concentrations (Fig. S2A). A total of 362 DEGs were common across all DCA-treated groups (Fig. 2A), and principal component analysis (PCA) revealed distinct transcriptional profiles among the different treatments (Fig. 2B). The expression of key virulence and regulatory genes, including *tcdA, tcdB, spo0A, fliC, ccpA, codY*, and *cwp19*, was significantly upregulated (Fig. 2C). We then focused on the D-proline reductase, a critical factor for CD colonization, growth, and energy metabolism. The expression of its constituent genes, *prdA, prdB*, and *prdC* [28], was upregulated in a dose-dependent manner (Fig. 2D). KEGG pathway enrichment analysis of the DEGs further indicated that DCA-induced transcriptional alterations were predominantly concentrated in amino acid metabolism pathways (Fig. 2E, F, and  G). To corroborate these findings, we performed metabolomic profiling of the co-culture supernatants. As DCA concentration increased, the relative abundance of organic acids, peptides, and lipids decreased, whereas that of steroids increased (Fig. 3A). Significant metabolic shifts were most pronounced in the high-DCA (CEH) group (Fig. S2B and C). As expected, the levels of DCA and other secondary bile acids were positively correlated with the exogenous DCA dose. Conversely, several amino acids, including tyrosine, DL-norvaline, L-glutamic acid, DL-arginine, DL-lysine, and N-acetyl-L-leucine, were inversely correlated with DCA concentration (Fig. S2C). The primary signature metabolites distinguishing the DCA-treated groups included 5-aminovaleric acid (5-APA), 4-aminobutyric acid, L-valine, and others (Fig. 3B, Fig. S2D). Pathway analysis of these differential metabolites highlighted significant alterations in D-amino acid metabolism, steroid hormone biosynthesis, and phenylalanine metabolism (Fig. 3C and D), which is highly consistent with the transcriptomic findings.

**Figure 2:**
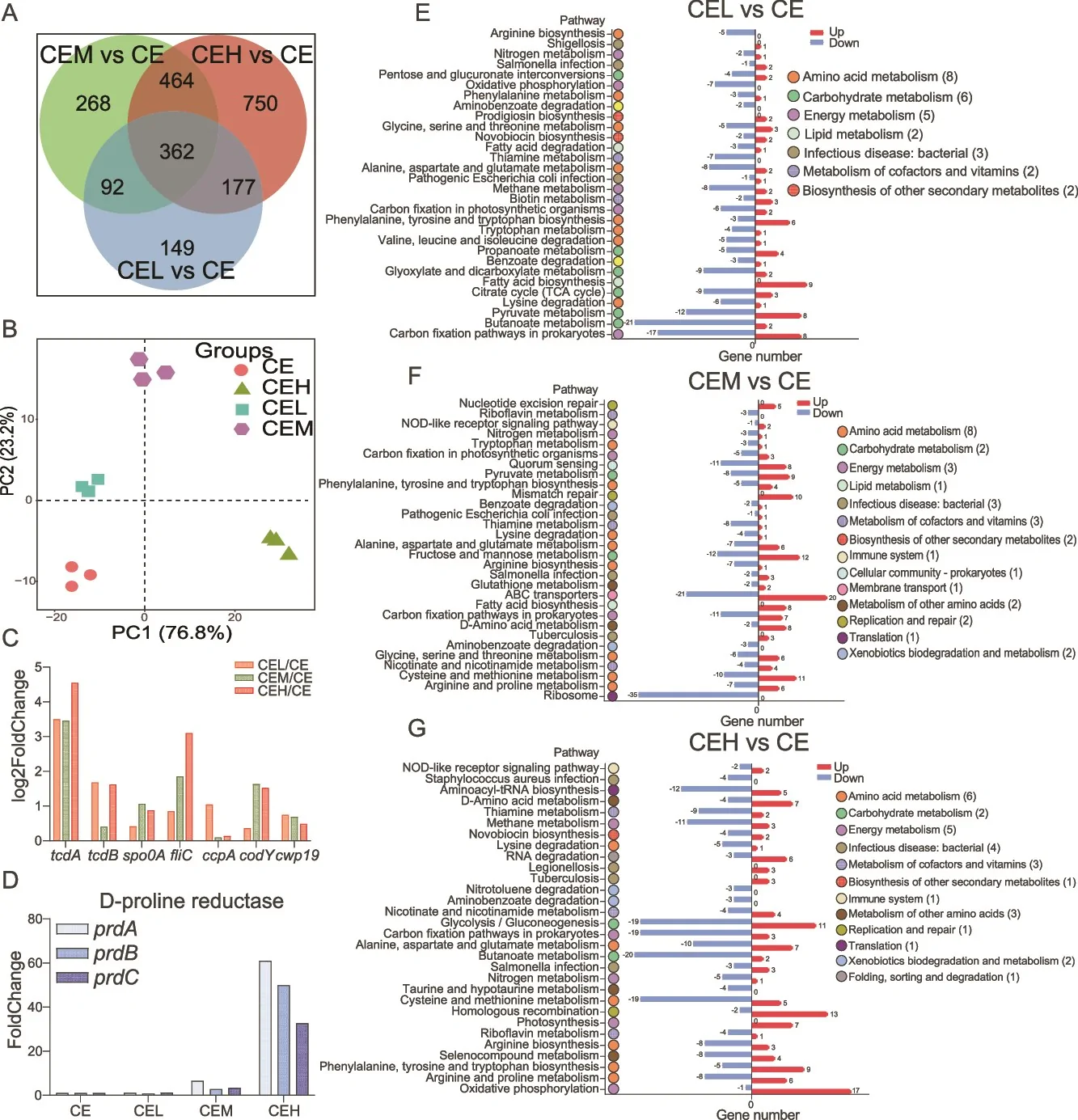
Transcriptomic analysis of *C. Difficile* (CD) in co-culture with *E. Coli* under DCA exposure. A. Common or unique differentially expressed genes (DEGs) across group comparisons. B. Principal component analysis (PCA) of gene expression profiles. C. The expression levels of core CD genes across different conditions. D. The expression levels of genes associated with D-proline reductase. E, F, G. KEGG pathway enrichment analysis for the DEGs, showing the top 30 most significantly enriched pathways ranked by *P* value. Note: For the KEGG plots, the x-axis represents the pathway name, and the numbers indicate the count of up-regulated and down-regulated genes within each pathway.

**Figure 3:**
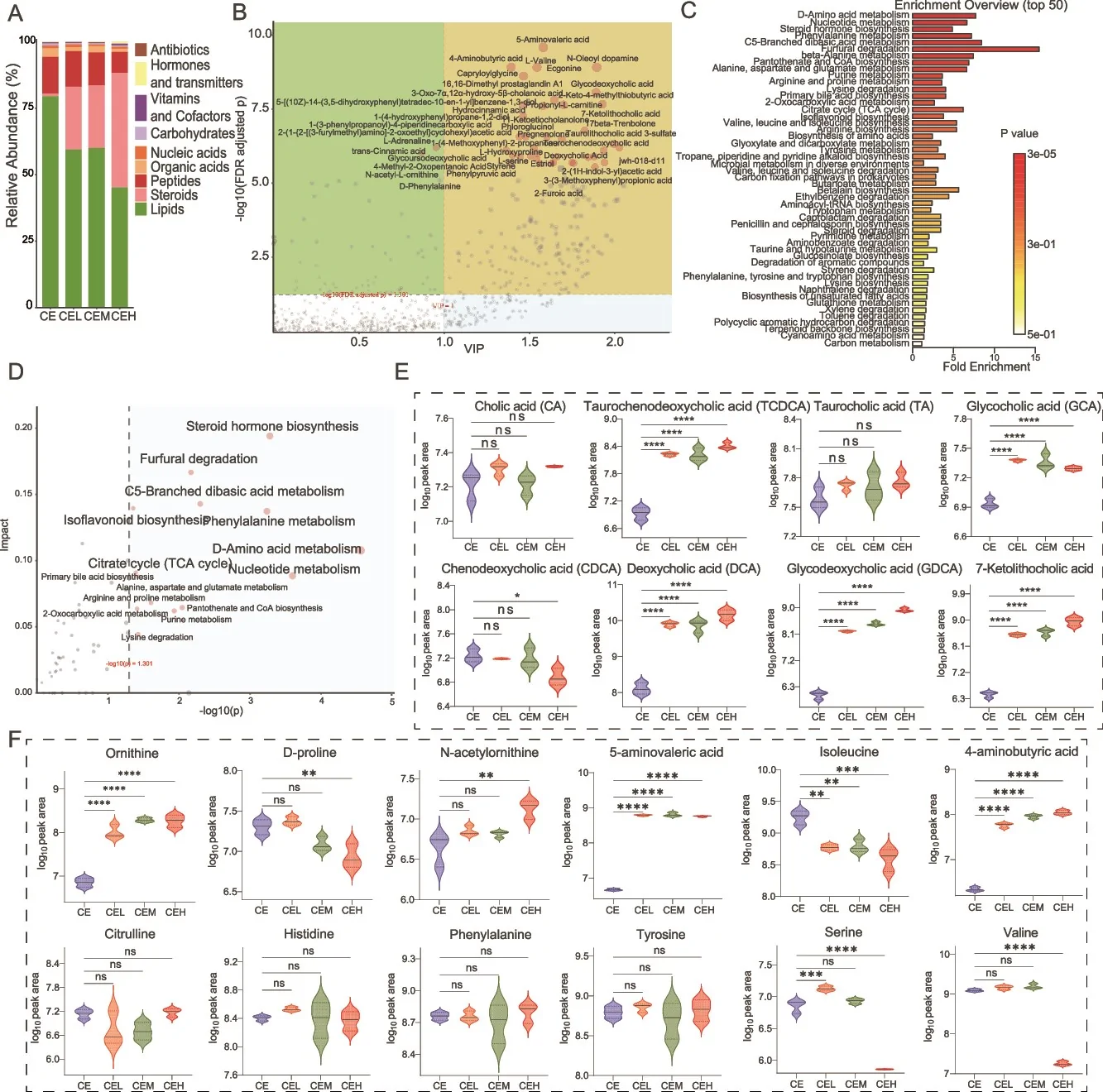
Metabolomic profiling of the *C. Difficile* (CD) and *E. Coli* (EC) co-culture system in the presence of DCA. A. Functional categorization of detected metabolites based on KEGG biological roles (br08001). B. Importance plot from Orthogonal Partial Least Squares-Discriminant Analysis (OPLS-DA). Each point represents a metabolite, plotted by its Variable Importance in Projection (VIP) score (x-axis) against its false discovery rate (FDR)-corrected *P*-value (log10-transformed, y-axis). C. Over-Representation Analysis (ORA) of metabolic pathways. The x-axis indicates the enrichment factor, calculated as the ratio of observed to expected metabolites in a given pathway. *P-*value magnitude is represented by color intensity. D. Combined ORA and pathway topology analysis. The x-axis shows the -log10 (*P*-value) from ORA, with the blue shaded area indicating statistical significance (*P* < .05). The y-axis represents the pathway impact score from topology analysis. E. Relative abundance of bile acid metabolites. F. Relative abundance of key amino acids involved in Stickland metabolism. Statistical significance in (E) and (F) was determined using Dunnett’s multiple comparisons test. For all panels, statistical significance was determined by Dunnett’s test: ns, not significant (*P* > .05); ^*^P < .05; ^**^P < .01; ^***^P < .001; ^****^P < .0001.

Given that bile acid composition and Stickland pathway amino acids are critical determinants of CD’s metabolic activity and pathogenicity [9, 17], we next investigated their specific dynamics. In DCA-supplemented co-cultures, the levels of both primary (e.g. TCDCA and GCA) and secondary (e.g. GDCA and 7-Ketolithocholic acid) bile acids were significantly elevated (Fig. 3E). Concurrently, we observed profound alterations in key Stickland pathway metabolites. Specifically, as DCA concentration rose, levels of ornithine, N-acetylornithine, 5-APA, and 4-aminobutyric acid increased, whereas D-proline and isoleucine levels sharply declined (Fig. 3F). These integrated findings indicate that whereas DCA presence broadly upregulates CD’s pathogenic transcriptome, the most pronounced metabolic shifts occur within the Stickland pathway, particularly involving the depletion of proline. This pattern implies that in a DCA-rich environment, CD and EC might establish a severe co-infection driven by a collaborative mechanism of amino acid provisioning, potentially wherein EC supplies the proline that CD catabolizes to enhance its growth and virulence.

### DCA potentiated the severity of *C. Difficile* and *E. Coli* co-infection in a murine model

Initially, we assessed the impact of a 7-day continuous intragastric administration of DCA on the expression levels of key bile acid-synthesizing enzymes in the livers of non-infected mice. The results revealed that, compared to the control group, CYP7A1 and CYP8B1 levels were significantly decreased in the high-dose DCA group, whereas CYP7B1 and CYP27A1 levels remained almost unaltered across all groups (Fig. S3A, B, C, and  D). To validate our *in vitro* findings, we employed a mouse model to assess the impact of DCA on CD and EC co-infection. At the experimental endpoint, mice in the CD group had the lowest survival rate (30%), compared to 90% in the EC group and 70% in the CE (CD + EC) group. The presence of DCA significantly accelerated mortality, especially in the high-DCA (CEH) group, where survival dropped below 50% (Fig. 4B). DCA intervention effectively prevented mortality in mice caused by single-strain bacterial infections. For instance, under continuous high-dose DCA administration, both CD- and EC-infected groups exhibited a final survival rate of 100% (Fig. S3E).

**Figure 4:**
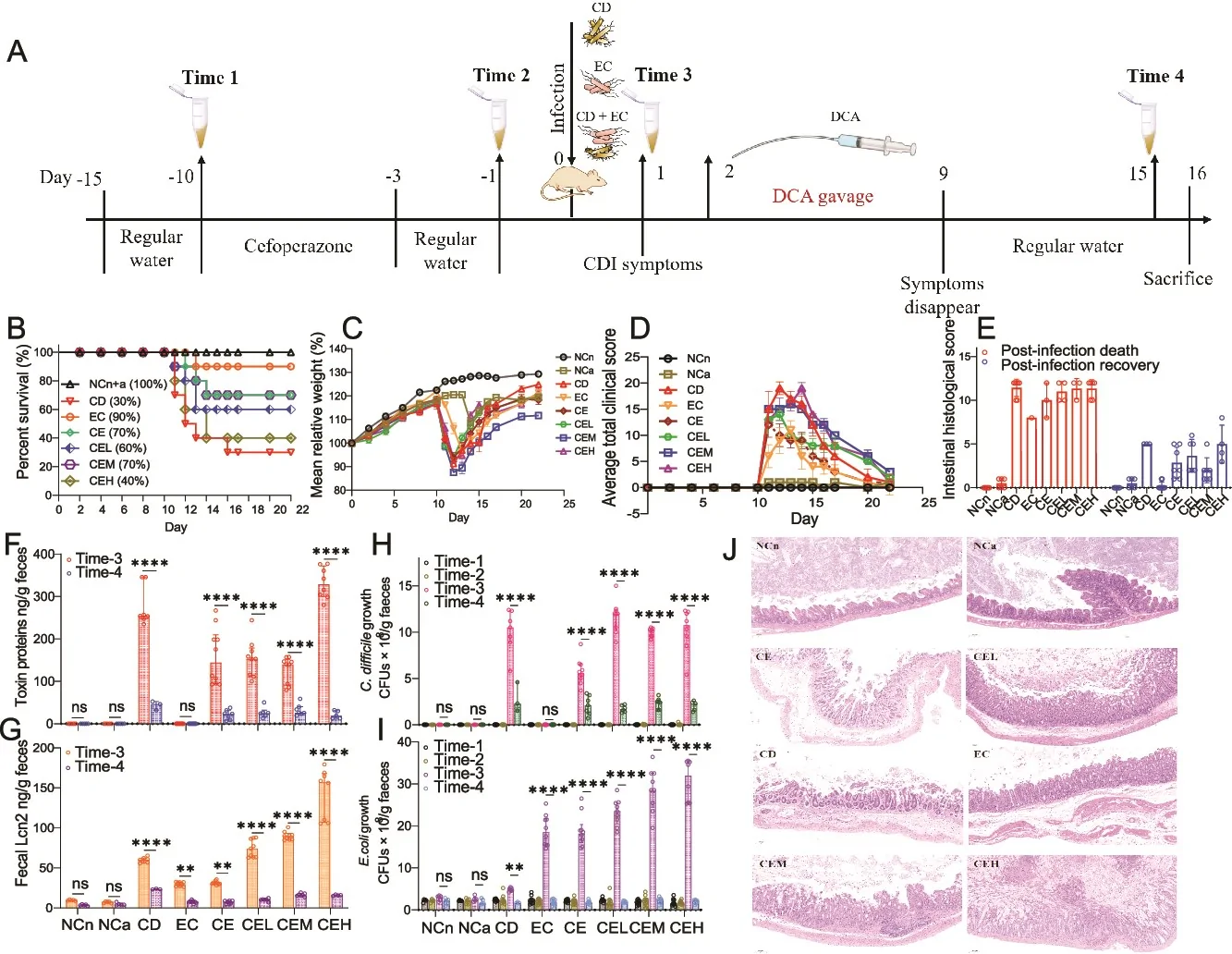
*E. Coli* (EC) exacerbates *C. Difficile* (CD) infection in a murine model in the presence of DCA. A. Schematic diagram of the experimental design and timeline. B. Kaplan–Meier survival curve of mice over the course of the infection. C. Changes in mean relative body weight. D. Mean total clinical score. E. Histopathological score of intestinal damage. F. Toxin protein levels in feces. G. Lcn2 levels in feces. H. Fecal burden of *C. Difficile*. I. Fecal burden of *E. Coli*. J. Representative H&E-stained cecal tissue sections. For all panels, statistical significance was determined by a two-tailed Student’s *t-*test: ns, not significant (*P* > .05); ^*^P < .05; ^**^P < .01; ^***^P < .001; ^****^P < .0001.

Correspondingly, all infected groups exhibited a marked loss in relative body weight, with the highest mortality occurring within the first four days post-infection. Surviving mice subsequently recovered to their pre-infection weights by the study’s conclusion (Fig. 4C). The average total clinical scores followed a trend identical to that of body weight loss (Fig. 4D). Despite the clear clinical differences, histopathological scoring of intestinal lesions revealed no significant differences among the infected groups (Fig. 4E). However, molecular and bacteriological analyses provided more sensitive metrics. At the peak of infection (T3), fecal levels of CD toxins and the host inflammatory marker lipocalin-2 (Lcn2) were elevated across all infected groups, exceeding 250 ng/g in the CD and CEH groups (Fig. 4F and G; Fig. S4A). Both markers declined by the recovery time point (T4) but remained higher than in the negative controls (NCn, NCa). Quantitative culture showed that fecal CD loads peaked at T3 and then declined, yet remained elevated above control levels at T4 (Fig. 4H). Similarly, EC loads, which were low pre-infection, increased significantly post-infection but returned to baseline levels by T4 in surviving mice (Fig. 4I). A positive correlation was observed between fecal DCA concentration and the levels of toxins, Lcn2, and bacterial loads at T3. Histological examination confirmed severe cecal damage in surviving or moribund mice, including edema and destruction of the villous architecture. By the endpoint (T4), surviving mice showed signs of tissue recovery, but this was incomplete, particularly in the CEL and CEH groups, where pathology scores did not return to control levels (Fig. 4J; Fig. S4B). In summary, these *in vivo* data demonstrate that continuous DCA administration exacerbates the severity of CD and EC co-infection, resulting in elevated mortality, heightened inflammatory responses, and impaired tissue recovery.

### DCA reshaped the gut microbiota and metabolome in mice during co-infection

We first assessed the impact of antibiotic pretreatment on the gut microbiota. Analysis at the T3 time point revealed that pretreatment led to a substantial depletion of commensal families, including *S24–7, Ruminococcaceae*, and *Bifidobacteriaceae*. Concurrently, it caused a rapid expansion and dominance of *Bacteroidaceae* and *Lactobacillaceae* (Fig. S5A, Fig. S6A). This perturbation was accompanied by a significant reduction in overall microbial diversity (alpha-diversity) and major alterations in community structure (beta-diversity) compared to untreated controls (NCa, NCb) (Fig. S5B and C, Fig. S6B and C). Post-infection, *Clostridiaceae* and *Enterococcaceae* proliferated across all infected groups (CD, EC, CE, CEL, CEM, CEH). *Enterobacteriaceae* and *Verrucomicrobiaceae* (*Akkermansia*) were significantly enriched specifically in the DCA-treated groups (CEL, CEM, CEH). The depletion of diversity and shifts in community structure induced by antibiotics persisted throughout the infection phase. By the experimental endpoint (T4), surviving mice began to show signs of microbiota recovery, with a concomitant reduction in intergroup compositional differences (Fig. S5B; Fig. S6B and C). Quantitative analysis confirmed that *C. difficile* and *E. coli* were highly enriched at T3, consistent with our data on viable bacteria on the plates (Fig. 4H and I), but *C. difficile* abundance declined in recovered mice at T4 (Fig. S5D and E, Fig. S6D and E). Machine learning approaches (Random Forest) and LEfSe analysis identified *C. difficile, Akkermansia muciniphila*, and *E. coli* as robust signature bacteria distinguishing the infection states (Fig. S5F and H). The functional abundance profiles inferred from metagenomic data showed high consistency with the observed taxonomic composition (Fig. S6F). KEGG pathway analysis further revealed that carbohydrate and amino acid metabolism were the two most significantly perturbed functional pathways among the groups (Fig. S5G).

Analysis of fecal samples collected at T3 identified 2499 metabolites, with “Lipids and lipid-like molecules” and “Organic acids and derivatives” constituting nearly half of the total. These categories encompassed the bile acids (BAs) and amino acids (Fig. S7A). The metabolite composition varied significantly across the different treatment groups (Fig. S7B). Given the critical roles of primary bile acids (PBAs) in promoting CD spore germination and secondary bile acids (SBAs) in inhibiting its growth [5, 6], we first characterized the BA pool. Antibiotic pretreatment alone led to a significant accumulation of PBAs (CA, CDCA, TCDCA, β-MCA) and SBAs (DCA, GDCA, iso-DCA, LCA, UCA) (Fig. S8A and B). Following infection, PBA levels (CA, GCA, CDCA) were further elevated in the DCA-treated groups (CEL, CEM, CEH) (Fig. 5A). Conversely, the levels of most SBAs, including DCA, decreased post-infection, approaching those of the negative control (NCn) (Fig. S8B). Compared to the CE group, DCA levels were lower in CEL, CEM, and CEH, whereas other SBAs like UCA and LCA were higher (Fig. 5B). These complex, non-linear changes in the BA profile suggested that other metabolic pathways were more indicative of the disease state. We therefore focused on amino acids within the Stickland pathway. Antibiotic pretreatment initially depleted several key amino acids (D-ornithine, D-proline, arginine), whereas elevating 5-aminovaleric acid (5-APA) (Fig. S8C). Post-infection, we observed a distinct metabolic signature in the high-mortality groups (CD, CEH): significant increases in D-ornithine, 5-APA, and N-acetylornithine, coupled with a sharp decrease in D-proline and isoleucine (Fig. 5C). This trend of D-proline depletion and 5-APA accumulation was consistent with our *in vitro* findings. Critically, whereas D-proline levels were elevated in the EC-only group, they were depleted in the CD and CE groups, an effect that was intensified by exogenous DCA (Fig. S8C). To formally test the observed metabolic relationships, we performed a correlation analysis. This revealed a strong positive correlation between CD abundance and the levels of 5-APA and N-acetylornithine, and a strong negative correlation with D-proline and isoleucine. The opposite pattern was observed for EC, which was positively correlated with D-proline and negatively with 5-APA (Fig. 5D). These data support our hypothesis that EC may serve as a supplier of proline in the gut, which is then catabolized by CD, resulting in the accumulation of the characteristic end-product, 5-APA.

**Figure 5:**
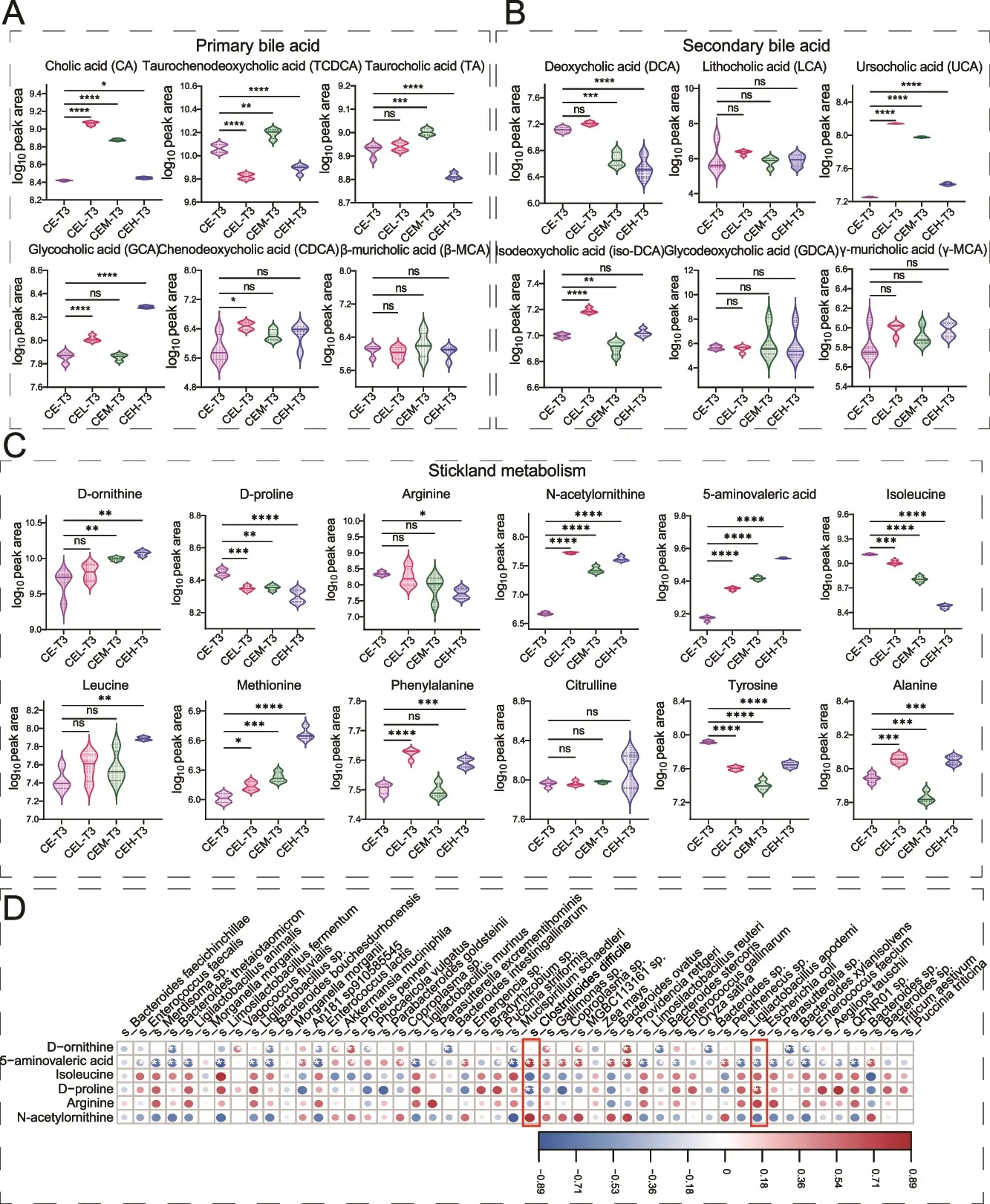
Fecal metabolomic profiling in the murine model of CDI. A. Relative abundance of primary bile acids. B. Relative abundance of secondary bile acids. C. Relative abundance of key amino acids involved in Stickland metabolism. D. Heatmap illustrating the correlation between microbial abundance and metabolite levels. In the heatmap, red and blue colors indicate positive and negative correlations, respectively, with color intensity representing the correlation strength. Asterisks (^*^) denote statistically significant microbe-metabolite pairs (*P* < 0.05). For all panels, statistical significance was determined by Dunnett’s test: ns, not significant (*P* > .05); ^*^  *P* < .05; ^**^  *P* < .01; ^***^P < .001; ^****^P < .0001.

### DCA promotes *E. Coli*-derived proline utilization to fuel *C. Difficile* growth and pathogenicity

To validate the proposed metabolic interaction, we first screened six key metabolites—arginine, 5-aminovaleric acid (5-APA), N-acetylornithine, isoleucine, D-proline, and D-ornithine—for their effects on bacterial growth. Whereas these metabolites had minimal impact on *E. coli* (EC), D-ornithine and D-proline significantly promoted the growth of *C. difficile* (CD) (Fig. 6A and B). We then focused on the Stickland pathway, where D-proline is catabolized by CD into 5-APA to generate energy and enhance virulence [4, 17]. We observed that in CD monoculture, DCA treatment paradoxically suppressed D-proline catabolism, leading to decreased 5-APA production (Fig. 6C and D). In a DCA-containing co-culture, this dynamic was reversed: D-proline was efficiently consumed by CD, leading to a reciprocal increase in 5-APA levels (Fig. 6C and D). This suggests that the presence of EC alleviates DCA’s inhibitory effect on CD’s D-proline metabolism. In BHI broth devoid of exogenous proline, EC was observed to secrete trace amounts of D-proline, a trend that was slightly enhanced by DCA treatment (Fig. 6E). Conversely, the presence of DCA elevated L-proline levels, indicating that EC possesses the capacity for L-proline biosynthesis and that this process is substantially promoted by DCA (Fig. 6F). Previous studies have demonstrated that proline racemase (regulated by *prdF*) is ubiquitous in CD [3, 28]. This enzyme catalyzes the isomerization of L-proline to D-proline, thereby facilitating the metabolic utilization of this amino acid by the pathogen. To uncover the molecular basis, we examined the expression of *prdF* and *prdB*, two key genes encoding the proline racemase and D-proline reductase. Whereas DCA suppressed *prdF* and *prdB* in CD monoculture, their expression was upregulated in the co-culture, restoring and even enhancing D-proline catabolism (Fig. 6G and H). This finding aligns with our transcriptomic data, which showed a dose-dependent upregulation of *prd* genes in the presence of DCA during co-culture (Fig. 2D). The essential role of this reductase was confirmed using a *prdB* knockout strain (△prdB) (Fig. S9A). Concurrently, this enhanced catabolism was linked to increased expression of virulence genes (*tcdA, tcdB*) and higher toxin production in CD (Fig. 6I, J, and  K). On the EC side, we found that DCA upregulated the expression levels of *proB* (proline synthesis) and *putP* (proline transport) [29, 30], explaining the increased L-proline production (Fig. 6L and M). Finally, we validated the universality of this interaction using different CD and EC strains, observing consistent trends in D-proline/5-APA dynamics and toxin production (Fig. 6N and O; Fig. S9B). Collectively, these results delineate a clear mechanism: in a DCA-rich environment, EC upregulates proline synthesis and transport, producing abundant L-proline. This amino acid is then efficiently converted to D-proline and catabolized by CD via a DCA-induced, EC-dependent upregulation of its racemase and D-proline reductase, ultimately fueling CD’s growth and enhancing its toxin production.

**Figure 6:**
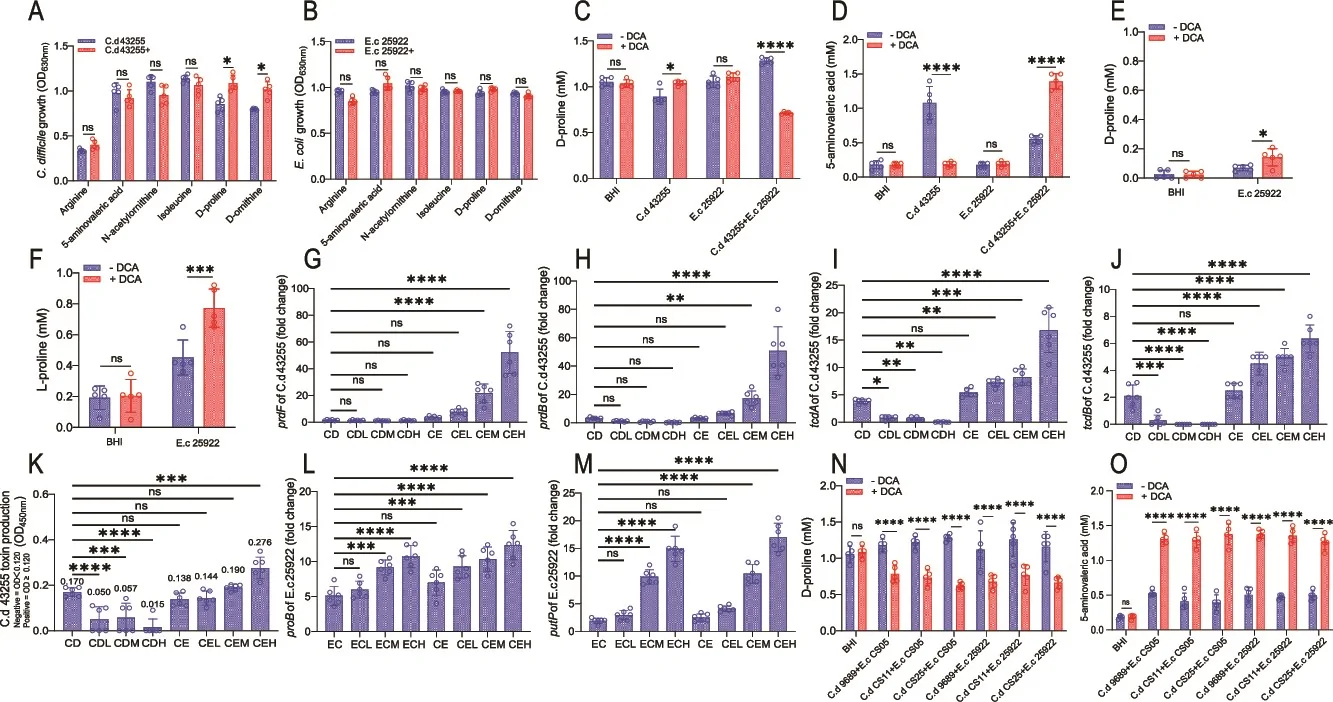
The role of D-proline in the *C. Difficile* (CD) and *E. Coli* (EC) interaction. A, B. Effects of specific metabolites on the growth of *C. d* 43 255 and *E. c* 25 922 with or without 1 mM DCA. C. Utilization of 1 mM D-proline by *C. d* 43 255 and *E. c* 25 922 in the presence or absence of 1 mM DCA. D. Quantification of 5-aminopentanoic acid (5-APA) production under different treatment conditions. Production of D-proline (E) and L-proline (F) by *E.c* 25 922 in standard BHI broth without exogenous proline, in the presence or absence of 1 mM DCA. G, H, I, J. Relative expression levels of *prdF, prdB, tcdA*, and *tcdB* in *C. d* 43 255 under various culture conditions. K. Toxin production in *C. d* 43 255 under different culture conditions. L, M. Relative expression levels of *proB* and *putP* in *E. c* 25 922 under various culture conditions. D-proline utilization (N) and 5-APA production (O) in co-cultures of different bacterial strains with or without 1 mM DCA. For Fig. 6A, B, C, D, E, F, N, and  O, statistical significance was determined by a two-tailed Student’s *t-*test; For Fig. 6G, H, I, J, K, L, and  M, statistical significance was determined by Dunnett’s test‌. ns, not significant (*P* > .05); ^*^P < .05; ^**^P < .01; ^***^P < .001; ^****^P < .0001.

To validate the generalizability of our findings, we employed two additional *C. difficile* strains with differing virulence: the low-virulence *C. d* 9689 and the moderately virulent *C. d* 630. In the *C. d* 9689 model, DCA was protective against CD-only infection but failed to prevent mortality when EC was present, with survival dropping to 60% (Fig. S10A). A similar trend was observed with the more virulent *C. d* 630 strain (Fig. S10B). These results confirm that, in a DCA environment, EC exacerbates the lethality of CDI across a spectrum of virulence levels. The critical role of D-proline reductase in this process was underscored by experiments using the *C. d* 630 (*△prdB*) knockout strain. The absence of the *prdB* gene, which encodes a key component of the reductase, led to a reduction in CD-induced mortality (Fig. S10B). This phenotype was directly linked to metabolic dysfunction, as fecal D-proline levels were significantly altered in the knockout strain compared to the wild-type (Fig. S10C and D). Furthermore, the inability to catabolize D-proline resulted in substantially lower toxin production in the *△prdB* strain during both mono- and co-infection (Fig. S10E and F). In contrast, wild-type *C. d* 9689 and *C. d* 630 strains efficiently utilized EC-derived proline, leading to a corresponding increase in toxin secretion. Furthermore, to assess whether the co-infection effects are generalizable across different *E. coli* strains, we established a murine co-infection model combining *C. d* 43 255 with two additional *E. coli* strains (*E. c* CS05 and *E. c* K-12). Under high-dose DCA treatment, the final survival rates of mice co-infected with *C. d* 43 255/*E. c* CS05 and *C. d* 43 255/*E. c* K-12 were 40% and 50%, respectively (Fig. S11A and B). Groups exhibiting higher mortality also displayed elevated CD toxin levels (Fig. S11C and D). Overall, the lethality trends induced by these alternative EC strains closely mirrored those of the *C. d* 43 255/*E. c* 25 922 co-infection model, indicating that the DCA-mediated exacerbation of CD and EC co-infection is conserved among different *E. coli* strains. Collectively, these *in vivo* validations confirm that the EC-provided proline, metabolized via the D-proline reductase pathway, is a universal and critical determinant of enhanced CD pathogenicity in the presence of DCA.

## Discussion

*Clostridioides difficile* is a ubiquitous anaerobic pathogen that colonizes the gastrointestinal tract. Perturbations to the gut microbiota, most due to antibiotic exposure, can trigger its pathogenic transformation, leading to the overproduction of toxins that inflict severe damage on the host intestinal mucosa. This clinical manifestation is defined as *C. difficile* infection (CDI) [4]. The current therapeutic landscape is stratified by disease recurrence: pCDI is typically managed with antibiotics, whereas fecal microbiota transplantation (FMT) has become the established treatment for rCDI [1]. Critically, the trajectory of the infection—whether towards resolution or exacerbation—is fundamentally governed by the state of the host’s intestinal ecosystem. This is primarily reflected in dysbiosis of the gut microbiota and concomitant shifts in the metabolomic profile [2, 5, 31]. Consequently, specific metabolites serve as key biomarkers for assessing treatment efficacy and host recovery. A hallmark of a healthy, restored gut ecosystem is the significant rebound of beneficial metabolites, such as short-chain fatty acids (SCFAs) like butyrate and secondary bile acids, deoxycholate (DCA) [9]. DCA is well-established for its potent inhibitory effects on CD, including suppression of vegetative growth, toxin production, and spore germination [6]. Paradoxically, however, elevated DCA levels do not invariably correlate with a favorable prognosis; in fact, they are observed in some cases of recurrent infection [32]. Given the demonstrable efficacy of DCA against CD, we hypothesized that additional, underlying mechanisms must contribute to this treatment-refractory phenotype. Indeed, a growing body of evidence implicates polymicrobial interactions as a key factor in CDI pathogenesis. Studies have identified symbiotic microbes, such as *Candida albicans, E. faecalis, Fusobacterium nucleatum*, and *B. thetaiotaomicron* that can facilitate treatment failure or recurrence [15, 17, 33, 34]. This suggests that CD may evade the harsh intestinal milieu, including high concentrations of antibiotics and secondary bile acids, through a collaborative relationship with specific microbes, thereby establishing a synergistic polymicrobial infection.

The paradigm of polymicrobial synergy in CDI is highly context-dependent, manifesting differently under various environmental pressures. For instance, CD-induced intestinal damage releases host hemoglobin, which fuels the rapid proliferation of *E. faecalis* [16]. In turn, *E. faecalis* provides CD with fermentable amino acids, creating a vicious cycle that exacerbates infection [17]. In an inflammatory milieu, *F. nucleatum* co-aggregates with CD on the mucosal layer, stimulating the production of extracellular polymeric substances and the formation of a protective biofilm that contributes to recurrence [33]. Similarly, under antibiotic stress, our prior work revealed that *B. thetaiotaomicron* forms a robust, VAN-induced biofilm with CD, significantly enhancing its drug resistance [15]. These examples illustrate that “poor treatment response” is often driven by such context-specific, collaborative interactions. This principle may extend to the DCA environment. Indeed, a metabolic cross-feeding loop has been described where CD-derived BAs are converted by *Clostridium scindens* into DCA, which in turn induces CD biofilm formation [13]. Building on this evidence, we hypothesize that specific microbes within the DCA environment interact with CD, and that this interaction is a prerequisite for the establishment of co-infection.

Beyond the well-characterized collaboration with *Enterococcus*, the *Enterobacteriaceae* family represents another cohort of microbes closely linked to CDI pathogenesis. Members of this family, particularly EC, are often enriched during active infection or in cases of poor treatment response [18, 19]. However, the ecological relationship between *Enterobacteriaceae* and CD remains poorly understood. Intriguingly, prior studies have shown that DCA significantly enhances the colonization capacity of EC without affecting its viability, whereas simultaneously exerting a potent inhibitory effect on CD [22]. This led us to hypothesize that a synergistic relationship between EC and CD could be established under DCA stress, enabling CD to circumvent DCA’ s antimicrobial activity. Indeed, our study confirmed this hypothesis: we first validated that DCA concentrations inhibitory to CD did not impede EC growth. Furthermore, whereas CD and EC exhibit a degree of mutualistic growth promotion *in vitro*, the presence of DCA intensified this interaction. This DCA-induced interaction culminated in the formation of a robust and dense symbiotic biofilm, which enhanced the survival of CD within the matrix (Fig. S1A). Concurrently, we observed a marked upregulation of genes associated with CD pathogenicity.

Integration of transcriptomic and metabolomic data revealed that DCA-mediated metabolic shifts were predominantly centered on the Stickland pathway, with D-proline emerging as a key differentially abundant metabolite. D-proline levels exhibited a dose-dependent response to DCA. This was mirrored at the transcriptional level in CD, where the expression of D-proline reductase, the principal enzyme for D-proline catabolism, was also upregulated in a DCA-dependent manner. These findings strongly suggest that D-proline serves as a critical energy substrate for CD survival and proliferation within this co-culture system. The catabolism of D-proline via redox reactions is known to fuel CD growth, toxin production, and sporulation [35, 36], with its end-product, 5-aminovaleric acid (5-APA), serving as a key biomarker for active infection [4]. In our study, 5-APA levels fluctuated inversely with D-proline, further confirming the active utilization of the latter by CD. The proline-dependent interaction between CD and EC involves a two-step process. First, substantial quantities of proline are generated within the co-culture. Second, DCA exposure enhances the efficiency of proline utilization by CD. We identified EC as the likely source of this proline, as EC is known to synthesize proline via the γ-glutamate kinase pathway (regulated by *proB*) [30] and import it through the *PutP* transporter [29]. We observed a clear dose-dependent upregulation of *proB* and *putP* in EC in response to DCA (Fig. 6L and M). This DCA-mediated, proline-fueled synergism was further validated in both *in vivo* and *in vitro* models using alternative CD and EC strains, confirming the central role of the D-proline reductase in CD pathogenicity. *E. coli* ATCC 25922 is widely used as a standard strain in various animal models and is generally regarded as a non-toxigenic, non-pathogenic strain. However, unexpected mortality was observed in the *E. coli* ATCC 25922 alone infection group during this study (Fig. 4B). Given the absence of mortality in *E. coli* ATCC 25922-treated mice across several other independent experiments (Fig. S10), we conclude that this isolated death is a sporadic event, most likely attributable to inter-individual variability or experimental handling errors.

Despite these compelling findings, our study has several limitations. It remains to be definitively proven whether the fluctuations in proline observed in the mouse model are exclusively driven by EC. The potential contribution of other gut microbes, particularly other *Enterobacteriaceae* (e.g. *Salmonella, K. pneumoniae*), also warrants investigation. Furthermore, the precise molecular mechanisms by which EC supplies and releases proline to CD under DCA stress require further elucidation. In conclusion, our data demonstrate that under DCA conditions, EC can supply proline to fuel CD metabolism, thereby enhancing its pathogenicity. The presence of EC may therefore be a critical factor underlying CDI recurrence in patients with high DCA levels. Moreover, this study suggests that targeting the abundance and composition of *Enterobacteriaceae*, such as EC, could represent a novel adjunctive strategy to improve the efficacy of conventional therapies, including antibiotics, probiotics, and FMT.

## Conclusions

Our study elucidates an interplay between CD and EC under deoxycholate (DCA) stress, characterized by the formation of a robust symbiotic biofilm that augments CD survival and toxin secretion. Through *in vitro* co-culture assays and murine models, we demonstrate that EC generates substantial quantities of proline in the presence of DCA. This metabolite is subsequently catabolized by the proline racemase and D-proline reductase in CD, thereby bolstering its environmental fitness and pathogenicity. These findings offer a mechanistic explanation for the paradoxical recurrence of *C. difficile* infection (CDI) in patients with elevated intestinal DCA levels.

## Supplementary Material

Supplementary_material_wrag249

## Data Availability

All sequencing data relevant to this study are deposited in NCBI, including transcriptome data (PRJNA903093), 16S rRNA amplicon sequencing data (PRJNA976903), and metagenomic data (PRJNA991623). The metabolomics data have been deposited in the OMIX, China National Center for Bioinformation/Beijing Institute of Genomics, Chinese Academy of Sciences (https://ngdc.cncb.ac.cn/omix/release/OMIX013182; https://ngdc.cncb.ac.cn/omix/release/OMIX013183).
