# Supplementary material for: Deoxycholate induces proline provision from Escherichia coli to enhance Clostridioides difficile virulence
Source: ISME J. 2026 Sep 17;20(1):wrag249. doi: 10.1093/ismejo/wrag249 (PMC13634199; doi:10.1093/ismejo/wrag249)
Supplement: Supplementary file 1 [file supplementary_material_wrag249.zip › Figure S4.pdf]

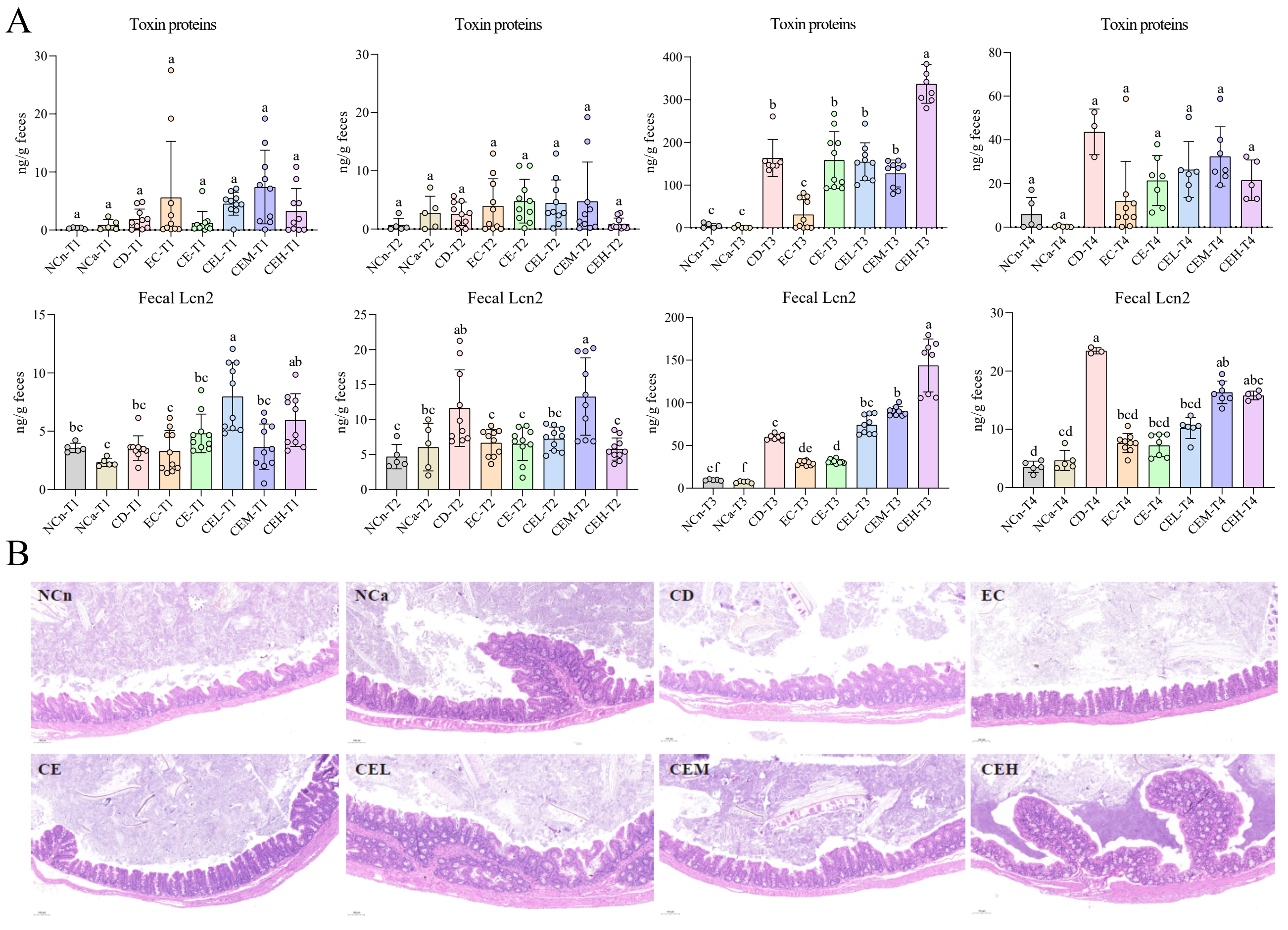
A
Toxin proteins
ng/g feces
NCn-T1
NCa-T1
CD-T1
EC-T1
CE-T1
CEL-T1
CEM-T1
CEH-T1
Toxin proteins
ng/g feces
NCn-T2
NCa-T2
CD-T2
EC-T2
CE-T2
CEL-T2
CEM-T2
CEH-T2
Toxin proteins
ng/g feces
NCn-T3
NCa-T3
CD-T3
EC-T3
CE-T3
CEL-T3
CEM-T3
CEH-T3
Toxin proteins
ng/g feces
NCn-T4
NCa-T4
CD-T4
EC-T4
CE-T4
CEL-T4
CEM-T4
CEH-T4
Fecal Lcn2
ng/g feces
Fecal Lcn2
ng/g feces
Fecal Lcn2
ng/g feces
Fecal Lcn2
ng/g feces
B
NCn
NCa
CD
EC
CE
CEL
CEM
CEH
