# Supplementary material for: Deoxycholate induces proline provision from Escherichia coli to enhance Clostridioides difficile virulence
Source: ISME J. 2026 Sep 17;20(1):wrag249. doi: 10.1093/ismejo/wrag249 (PMC13634199; doi:10.1093/ismejo/wrag249)
Supplement: Supplementary file 1 [file supplementary_material_wrag249.zip › Figure S5.pdf]

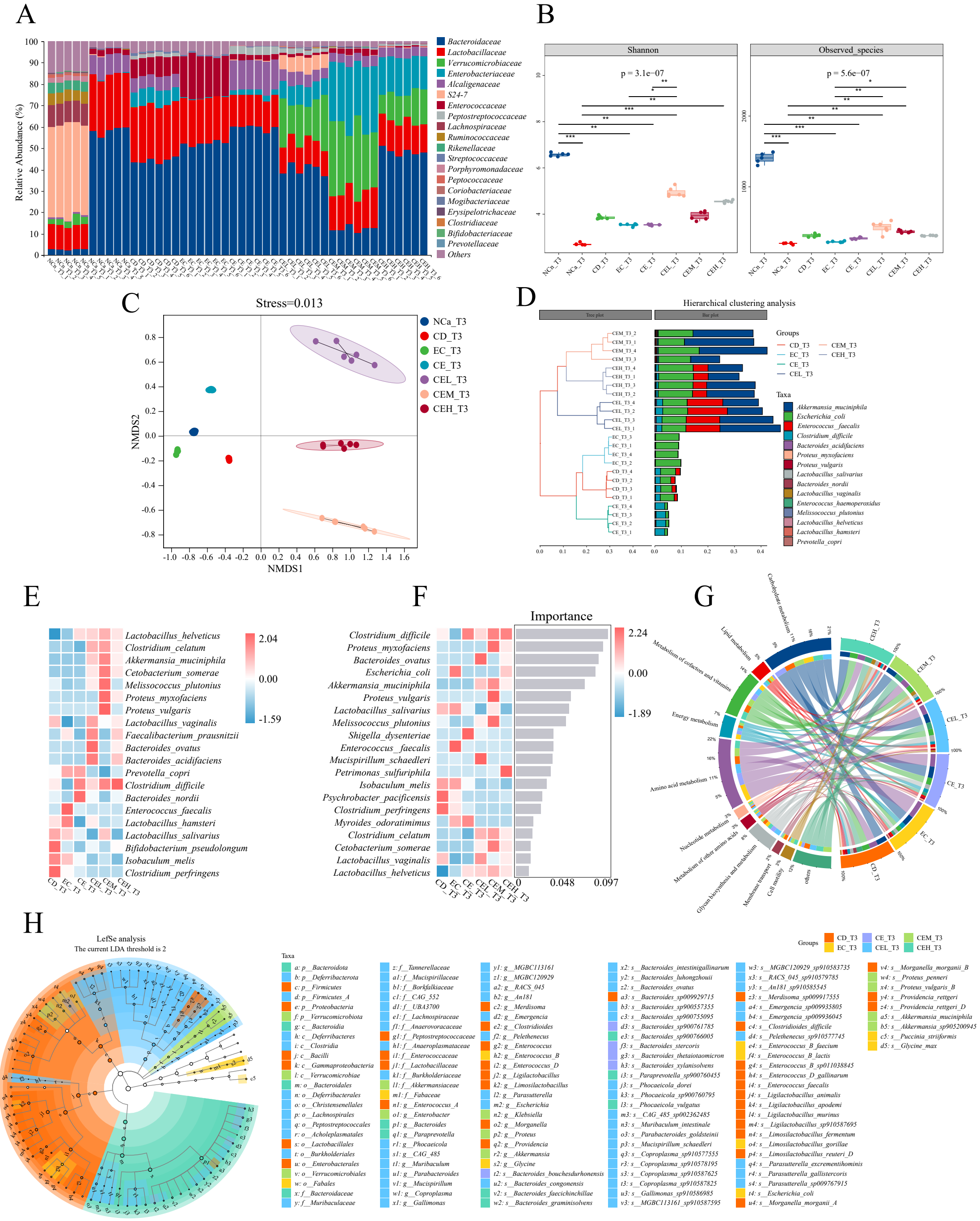
A
Relative Abundance (%)
Bacteroidaceae
Lactobacillaceae
Verrucomicrobiaceae
Enterobacteriaceae
Alcaligenaceae
S24-7
Enterococcaceae
Peptostreptococcaceae
Lachnospiraceae
Ruminococcaceae
Rikenellaceae
Streptococcaceae
Porphyromonadaceae
Peptococcaceae
Coriobacteriaceae
Mogibacteriaceae
Erysipelotrichaceae
Clostridiaceae
Bifidobacteriaceae
Prevotellaceae
Others
B
Shannon
p = 3.1e−07
Observed_species
p = 5.6e−07
NCn_T3
NCa_T3
CD_T3
EC_T3
CE_T3
CEL_T3
CEM_T3
CEH_T3
C
Stress=0.013
NMDS1
NMDS2
NCa_T3
CD_T3
EC_T3
CE_T3
CEL_T3
CEM_T3
CEH_T3
D
Hierarchical clustering analysis
Tree plot
Bar plot
Groups
Taxa
Akkermansia_muciniphila
Escherichia_coli
Enterococcus_faecalis
Clostridium_difficile
Bacteroides_acidifaciens
Proteus_myxofaciens
Proteus_vulgaris
Lactobacillus_salivarius
Bacteroides_nordii
Lactobacillus_vaginalis
Enterococcus_haemoperoxidus
Melissococcus_plutonius
Lactobacillus_helveticus
Lactobacillus_hamsteri
Prevotella_copri
E
Lactobacillus_helveticus
Clostridium_celatum
Akkermansia_muciniphila
Cetobacterium_somerae
Melissococcus_plutonius
Proteus_myxofaciens
Proteus_vulgaris
Lactobacillus_vaginalis
Faecalibacterium_prausnitzii
Bacteroides_ovatus
Bacteroides_acidifaciens
Prevotella_copri
Clostridium_difficile
Bacteroides_nordii
Enterococcus_faecalis
Lactobacillus_hamsteri
Lactobacillus_salivarius
Bifidobacterium_pseudolongum
Isobaculum_melis
Clostridium_perfringens
2.04
0.00
-1.59
F
Importance
Clostridium_difficile
Proteus_myxofaciens
Bacteroides_ovatus
Escherichia_coli
Akkermansia_muciniphila
Proteus_vulgaris
Lactobacillus_salivarius
Melissococcus_plutonius
Shigella_dysenteriae
Enterococcus_faecalis
Mucispirillum_schaedleri
Petrimonas_sulfuriphila
Isobaculum_melis
Psychrobacter_pacificensis
Clostridium_perfringens
Myroides_odoratimimus
Clostridium_celatum
Cetobacterium_somerae
Lactobacillus_vaginalis
Lactobacillus_helveticus
0
0.048
0.097
2.24
0.00
-1.89
G
Carbohydrate metabolism
Lipid metabolism
Metabolism of cofactors and vitamins
Energy metabolism
Amino acid metabolism
Nucleotide metabolism
Metabolism of other amino acids
Glycan biosynthesis and metabolism
Membrane transport
Cell motility
others
H
LefSe analysis
The current LDA threshold is 2
Groups
Taxa
a: p__Bacteroidota
b: p__Deferribacterota
c: p__Firmicutes
d: p__Firmicutes_A
e: p__Proteobacteria
f: p__Verrucomicrobiota
g: c__Bacteroidia
h: c__Deferribacteres
i: c__Clostridia
j: c__Bacilli
k: c__Gammaproteobacteria
l: c__Verrucomicrobiae
m: o__Bacteroidales
n: o__Deferribacterales
o: o__Christensenellales
p: o__Lachnospirales
q: o__Peptostreptococcales
r: o__Acholeplasmatales
s: o__Lactobacillales
t: o__Burkholderiales
u: o__Enterobacterales
v: o__Verrucomicrobiales
w: o__Fabales
x: f__Bacteroidaceae
y: f__Muribaculaceae
z: f__Tannerellaceae
a1: f__Mucispirillaceae
b1: f__Borkfalkiaceae
c1: f__CAG_552
d1: f__UBA3700
e1: f__Lachnospiraceae
f1: f__Anaerovoracaceae
g1: f__Peptostreptococcaceae
h1: f__Anaeroplasmataceae
i1: f__Enterococcaceae
j1: f__Lactobacillaceae
k1: f__Burkholderiaceae
l1: f__Akkermansiaceae
m1: f__Fabaceae
n1: g__Enterococcus_A
o1: g__Enterobacter
p1: g__Bacteroides
q1: g__Paraprevotella
r1: g__Phocaeicola
s1: g__CAG_485
t1: g__Muribaculum
u1: g__Parabacteroides
v1: g__Mucispirillum
w1: g__Coproplasma
x1: g__Gallimonas
y1: g__MGBC113161
z1: g__MGBC120929
a2: g__RACS_045
b2: g__An181
c2: g__Merdisoma
d2: g__Emergencia
e2: g__Clostridioides
f2: g__Pelethenecus
g2: g__Enterococcus
h2: g__Enterococcus_B
i2: g__Enterococcus_D
j2: g__Ligilactobacillus
k2: g__Limosilactobacillus
l2: g__Parasutterella
m2: g__Escherichia
n2: g__Klebsiella
o2: g__Morganella
p2: g__Proteus
q2: g__Providencia
r2: g__Akkermansia
s2: g__Glycine
t2: s__Bacteroides_bouchesdurhonensis
u2: s__Bacteroides_congonensis
v2: s__Bacteroides_faecichinchillae
w2: s__Bacteroides_graminisolvens
x2: s__Bacteroides_intestinigallinarum
y2: s__Bacteroides_luhongzhouii
z2: s__Bacteroides_ovatus
a3: s__Bacteroides_sp009929715
b3: s__Bacteroides_sp900557355
c3: s__Bacteroides_sp900755095
d3: s__Bacteroides_sp900761785
e3: s__Bacteroides_sp900766005
f3: s__Bacteroides_stercoris
g3: s__Bacteroides_thetaiotaomicron
h3: s__Bacteroides_xylanisolvens
i3: s__Paraprevotella_sp900760455
j3: s__Phocaeicola_dorei
k3: s__Phocaeicola_sp900760795
l3: s__Phocaeicola_vulgatus
m3: s__CAG_485_sp002362485
n3: s__Muribaculum_intestinale
o3: s__Parabacteroides_goldsteinii
p3: s__Mucispirillum_schaedleri
q3: s__Coproplasma_sp910577555
r3: s__Coproplasma_sp910578195
s3: s__Coproplasma_sp910587625
t3: s__Coproplasma_sp910587825
u3: s__Gallimonas_sp910586985
v3: s__MGBC113161_sp910587595
w3: s__MGBC120929_sp910583735
x3: s__RACS_045_sp910579785
y3: s__An181_sp910585545
z3: s__Merdisoma_sp009917555
a4: s__Emergencia_sp009935805
b4: s__Emergencia_sp009936045
c4: s__Clostridioides_difficile
d4: s__Pelethenecus_sp910577745
e4: s__Enterococcus_B_faecium
f4: s__Enterococcus_B_lactis
g4: s__Enterococcus_B_sp011038845
h4: s__Enterococcus_D_gallinarum
i4: s__Enterococcus_faecalis
j4: s__Ligilactobacillus_animalis
k4: s__Ligilactobacillus_apodemi
l4: s__Ligilactobacillus_murinus
m4: s__Ligilactobacillus_sp910587695
n4: s__Limosilactobacillus_fermentum
o4: s__Limosilactobacillus_gorillae
p4: s__Limosilactobacillus_reuteri_D
q4: s__Parasutterella_excrementihominis
r4: s__Parasutterella_gallistercoris
s4: s__Parasutterella_sp009767915
t4: s__Escherichia_coli
u4: s__Morganella_morganii_A
v4: s__Morganella_morganii_B
w4: s__Proteus_penneri
x4: s__Proteus_vulgaris_B
y4: s__Providencia_rettgeri
z4: s__Providencia_rettgeri_D
a5: s__Akkermansia_muciniphila
b5: s__Akkermansia_sp905200945
c5: s__Puccinia_striiformis
d5: s__Glycine_max
