# Supplementary material for: Deoxycholate induces proline provision from Escherichia coli to enhance Clostridioides difficile virulence
Source: ISME J. 2026 Sep 17;20(1):wrag249. doi: 10.1093/ismejo/wrag249 (PMC13634199; doi:10.1093/ismejo/wrag249)
Supplement: Supplementary file 1 [file supplementary_material_wrag249.zip › Figure S6.pdf]

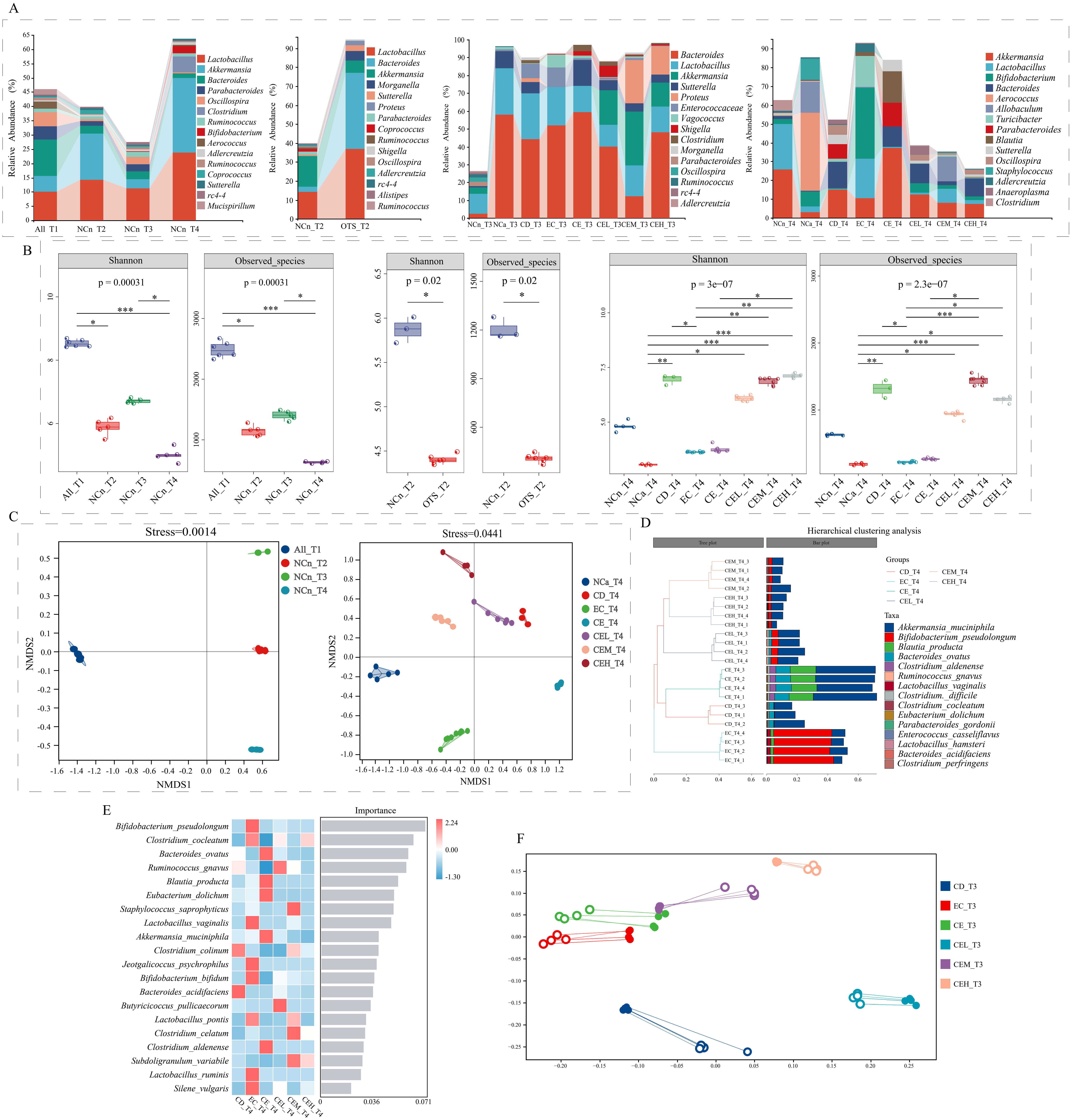
A
Relative Abundance (%)
All_T1
NCn_T2
NCn_T3
NCn_T4
Lactobacillus
Akkermansia
Bacteroides
Parabacteroides
Oscillospira
Clostridium
Ruminococcus
Bifidobacterium
Aerococcus
Adlercreutzia
Ruminococcus
Coprococcus
Sutterella
rc4-4
Mucispirillum
OTS_T2
Lactobacillus
Bacteroides
Akkermansia
Morganella
Sutterella
Proteus
Parabacteroides
Coprococcus
Ruminococcus
Shigella
Oscillospira
Adlercreutzia
rc4-4
Alistipes
Ruminococcus
NCn_T3
NCa_T3
CD_T3
EC_T3
CE_T3
CEL_T3
CEM_T3
CEH_T3
Bacteroides
Lactobacillus
Akkermansia
Sutterella
Proteus
Enterococcaceae
Vagococcus
Shigella
Clostridium
Morganella
Parabacteroides
Oscillospira
Ruminococcus
rc4-4
Adlercreutzia
NCn_T4
NCa_T4
CD_T4
EC_T4
CE_T4
CEL_T4
CEM_T4
CEH_T4
Akkermansia
Lactobacillus
Bifidobacterium
Bacteroides
Aerococcus
Allobaculum
Turicibacter
Parabacteroides
Blautia
Sutterella
Oscillospira
Staphylococcus
Adlercreutzia
Anaeroplasma
Clostridium
B
Shannon
p = 0.00031
Observed_species
p = 0.00031
Shannon
p = 0.02
Observed_species
p = 0.02
Shannon
p = 3e−07
Observed_species
p = 2.3e−07
C
Stress=0.0014
All_T1
NCn_T2
NCn_T3
NCn_T4
NMDS1
NMDS2
Stress=0.0441
NCa_T4
CD_T4
EC_T4
CE_T4
CEL_T4
CEM_T4
CEH_T4
D
Hierarchical clustering analysis
Tree plot
Bar plot
Groups
CD_T4
EC_T4
CE_T4
CEL_T4
CEM_T4
CEH_T4
Taxa
Akkermansia_muciniphila
Bifidobacterium_pseudolongum
Blautia_producta
Bacteroides_ovatus
Clostridium_aldenense
Ruminococcus_gnavus
Lactobacillus_vaginalis
Clostridium._difficile
Clostridium_cocleatum
Eubacterium_dolichum
Parabacteroides_gordonii
Enterococcus_casseliflavus
Lactobacillus_hamsteri
Bacteroides_acidifaciens
Clostridium_perfringens
E
Importance
Bifidobacterium_pseudolongum
Clostridium_cocleatum
Bacteroides_ovatus
Ruminococcus_gnavus
Blautia_producta
Eubacterium_dolichum
Staphylococcus_saprophyticus
Lactobacillus_vaginalis
Akkermansia_muciniphila
Clostridium_colinum
Jeotgalicoccus_psychrophilus
Bifidobacterium_bifidum
Bacteroides_acidifaciens
Butyricicoccus_pullicaecorum
Lactobacillus_pontis
Clostridium_celatum
Clostridium_aldenense
Subdoligranulum_variabile
Lactobacillus_ruminis
Silene_vulgaris
CD_T4
EC_T4
CE_T4
CEL_T4
CEM_T4
CEH_T4
2.24
0.00
-1.30
0
0.036
0.071
F
CD_T3
EC_T3
CE_T3
CEL_T3
CEM_T3
CEH_T3
