# Supplementary material for: Deoxycholate induces proline provision from Escherichia coli to enhance Clostridioides difficile virulence
Source: ISME J. 2026 Sep 17;20(1):wrag249. doi: 10.1093/ismejo/wrag249 (PMC13634199; doi:10.1093/ismejo/wrag249)
Supplement: Supplementary file 1 [file supplementary_material_wrag249.zip › Figure S7.pdf]

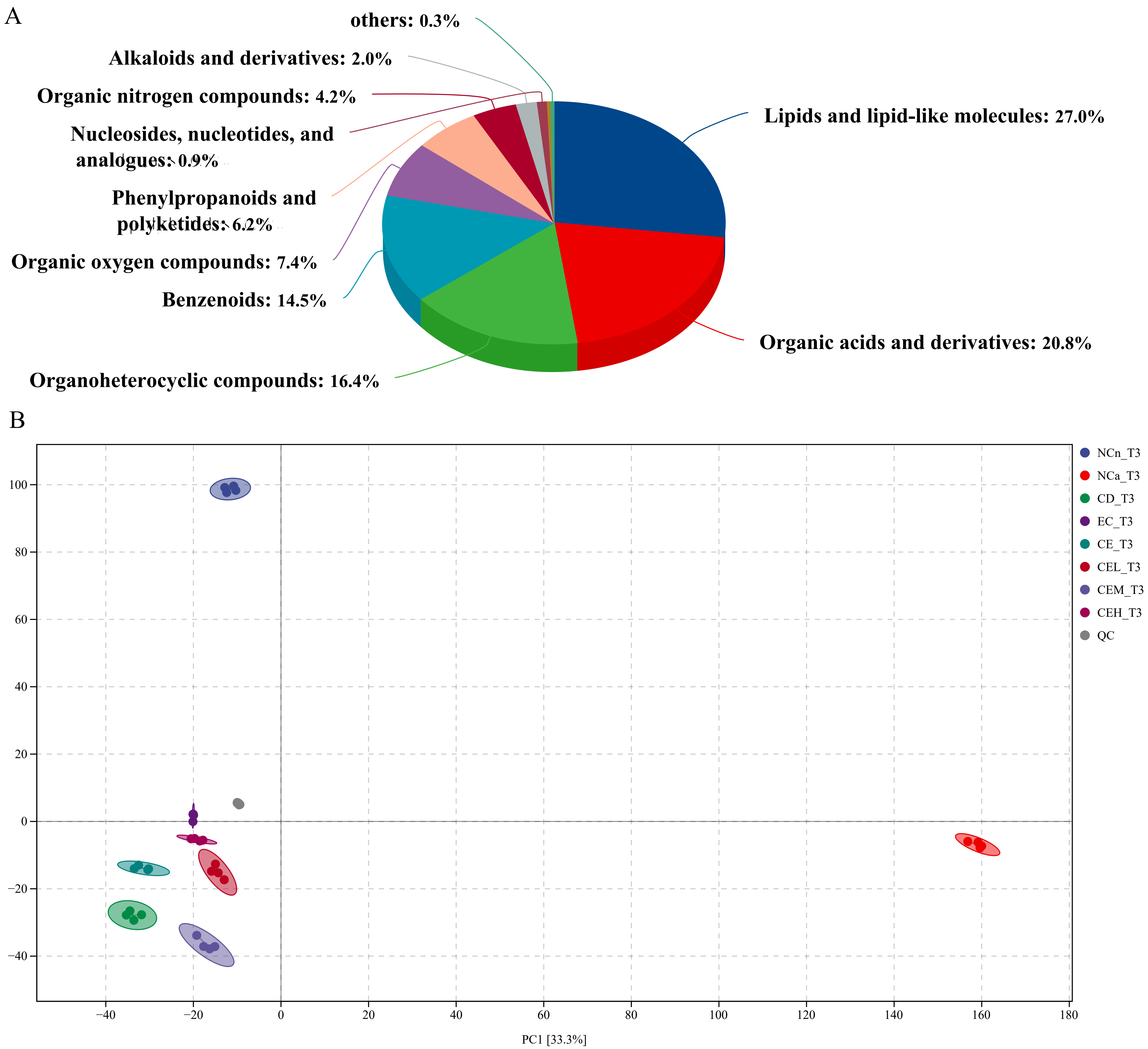
A
others: 0.3%
Alkaloids and derivatives: 2.0%
Organic nitrogen compounds: 4.2%
Nucleosides, nucleotides, and analogues: 0.9%
Phenylpropanoids and polyketides: 6.2%
Organic oxygen compounds: 7.4%
Benzenoids: 14.5%
Organoheterocyclic compounds: 16.4%
Lipids and lipid-like molecules: 27.0%
Organic acids and derivatives: 20.8%
B
NCn_T3
NCa_T3
CD_T3
EC_T3
CE_T3
CEL_T3
CEM_T3
CEH_T3
QC
PC2 [14.3%]
PC1 [33.3%]
100
80
60
40
20
0
−20
−40
−40
−20
0
20
40
60
80
100
120
140
160
180
