# Supplementary material for: Deoxycholate induces proline provision from Escherichia coli to enhance Clostridioides difficile virulence
Source: ISME J. 2026 Sep 17;20(1):wrag249. doi: 10.1093/ismejo/wrag249 (PMC13634199; doi:10.1093/ismejo/wrag249)
Supplement: Supplementary file 1 [file supplementary_material_wrag249.zip › Figure S8.pdf]

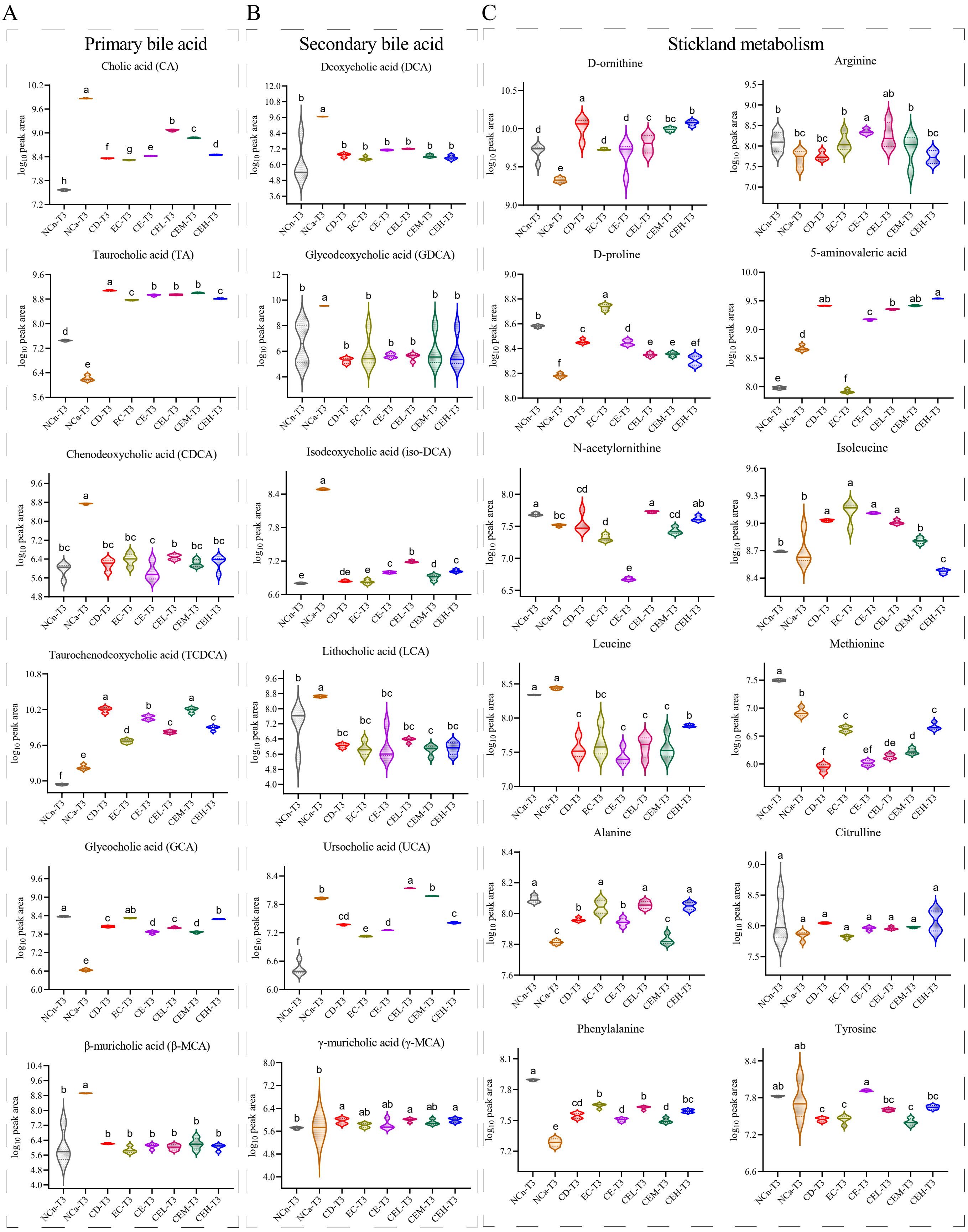

A
Primary bile acid
Cholic acid (CA)
Taurocholic acid (TA)
Chenodeoxycholic acid (CDCA)
Taurochenodeoxycholic acid (TCDCA)
Glycocholic acid (GCA)
β-muricholic acid (β-MCA)
B
Secondary bile acid
Deoxycholic acid (DCA)
Glycodeoxycholic acid (GDCA)
Isodeoxycholic acid (iso-DCA)
Lithocholic acid (LCA)
Ursocholic acid (UCA)
γ-muricholic acid (γ-MCA)
C
Stickland metabolism
D-ornithine
Arginine
D-proline
5-aminovaleric acid
N-acetylornithine
Isoleucine
Leucine
Methionine
Alanine
Citrulline
Phenylalanine
Tyrosine
log10 peak area
NCn-T3
NCa-T3
CD-T3
EC-T3
CE-T3
CEL-T3
CEM-T3
CEH-T3
