# Supplementary material for: Deoxycholate induces proline provision from Escherichia coli to enhance Clostridioides difficile virulence
Source: ISME J. 2026 Sep 17;20(1):wrag249. doi: 10.1093/ismejo/wrag249 (PMC13634199; doi:10.1093/ismejo/wrag249)
Supplement: Supplementary file 1 [file supplementary_material_wrag249.zip › Figure S9.pdf]

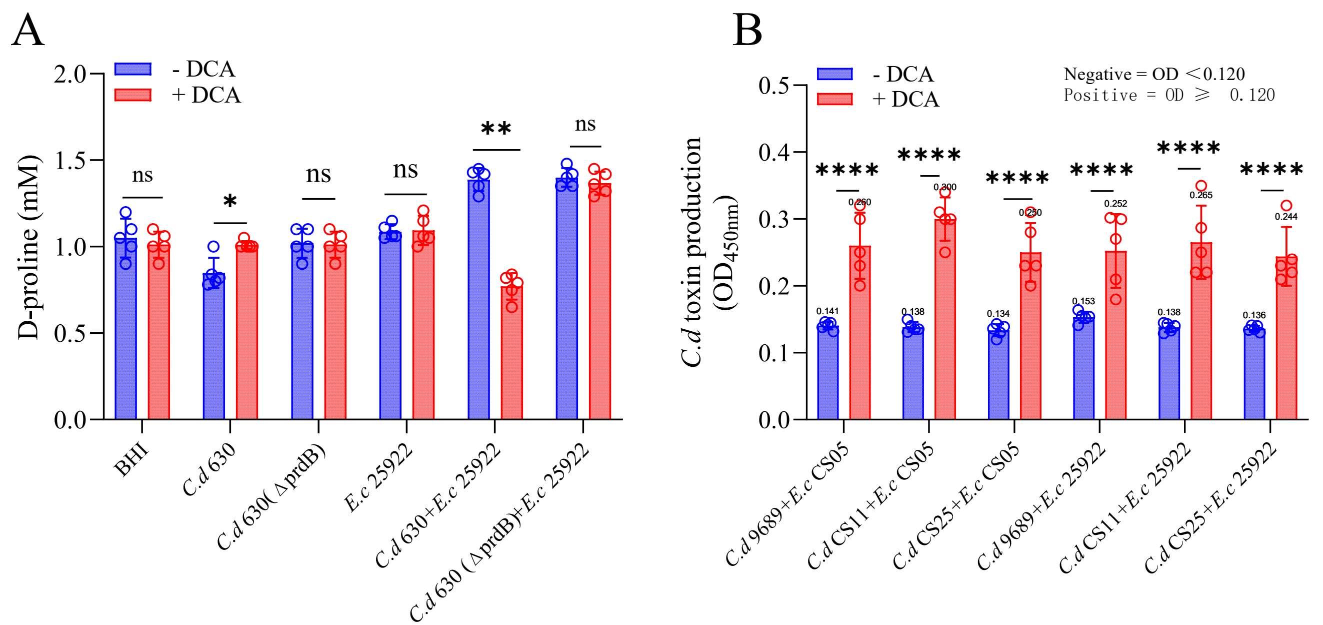
A
- DCA
+ DCA
D-proline (mM)
2.0
1.5
1.0
0.5
0.0
ns
*
ns
ns
**
ns
BHI
C.d 630
C.d 630(ΔprdB)
E.c 25922
C.d 630+E.c 25922
C.d 630 (ΔprdB)+E.c 25922
B
- DCA
+ DCA
Negative = OD <0.120
Positive = OD ⩾ 0.120
C.d toxin production (OD450nm)
0.5
0.4
0.3
0.2
0.1
0.0
****
****
****
****
****
****
0.141
0.260
0.138
0.300
0.134
0.250
0.153
0.252
0.138
0.265
0.136
0.244
C.d 9689+E.c CS05
C.d CS11+E.c CS05
C.d CS25+E.c CS05
C.d 9689+E.c 25922
C.d CS11+E.c 25922
C.d CS25+E.c 25922
