# Supplementary material for: Deoxycholate induces proline provision from Escherichia coli to enhance Clostridioides difficile virulence
Source: ISME J. 2026 Sep 17;20(1):wrag249. doi: 10.1093/ismejo/wrag249 (PMC13634199; doi:10.1093/ismejo/wrag249)
Supplement: Supplementary file 1 [file supplementary_material_wrag249.zip › Figure S10.pdf]

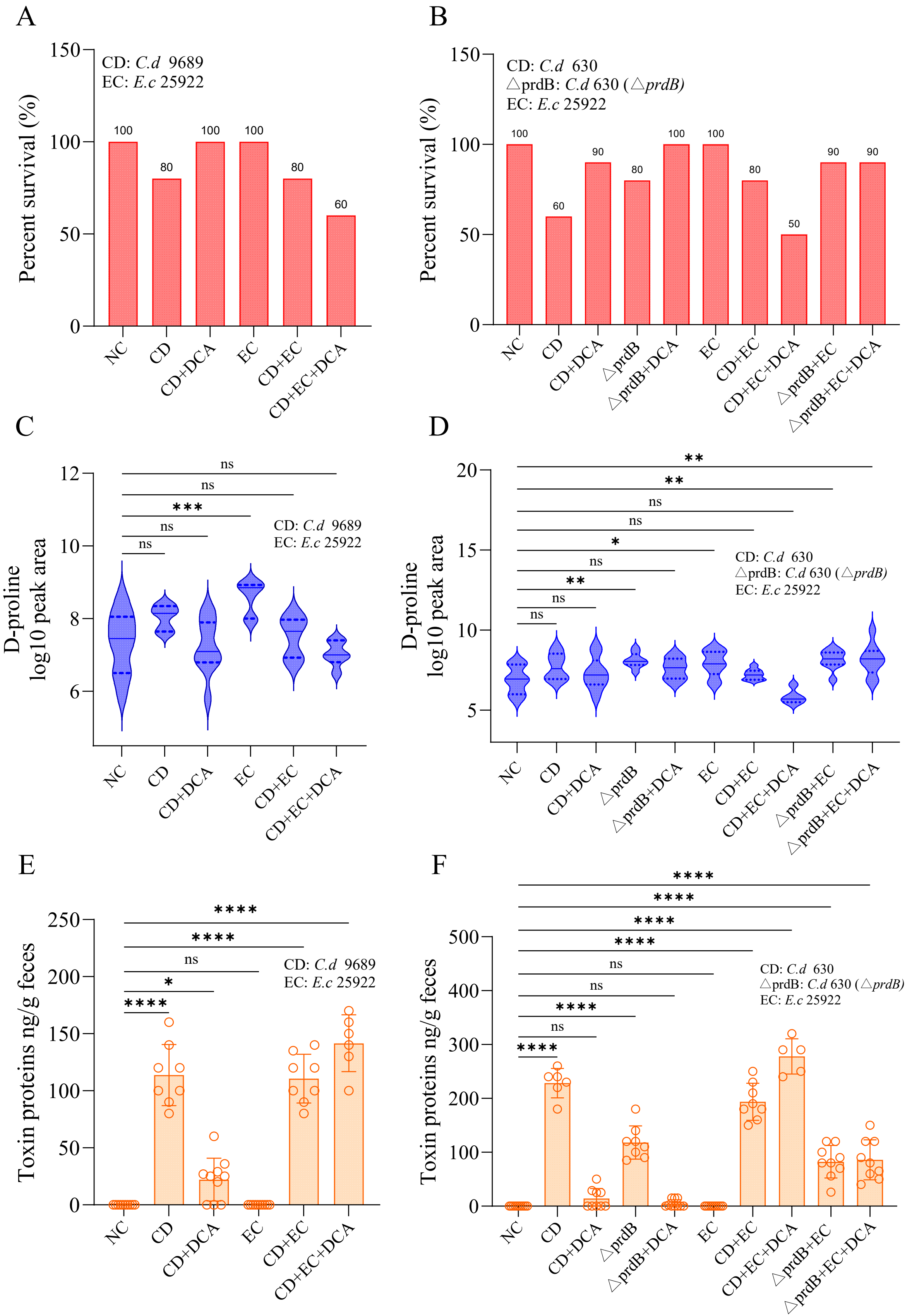
A
Percent survival (%)
CD: C.d 9689
EC: E.c 25922
100
80
100
100
80
60
NC
CD
CD+DCA
EC
CD+EC
CD+EC+DCA
B
Percent survival (%)
CD: C.d 630
△prdB: C.d 630 (△prdB)
EC: E.c 25922
100
60
90
80
100
100
80
50
90
90
NC
CD
CD+DCA
△prdB
△prdB+DCA
EC
CD+EC
CD+EC+DCA
△prdB+EC
△prdB+EC+DCA
C
D-proline
log10 peak area
CD: C.d 9689
EC: E.c 25922
ns
ns
***
ns
ns
D
D-proline
log10 peak area
CD: C.d 630
△prdB: C.d 630 (△prdB)
EC: E.c 25922
**
**
ns
ns
*
ns
**
ns
ns
E
Toxin proteins ng/g feces
CD: C.d 9689
EC: E.c 25922
****
****
ns
*
****
F
Toxin proteins ng/g feces
CD: C.d 630
△prdB: C.d 630 (△prdB)
EC: E.c 25922
****
****
****
****
ns
ns
****
ns
****
