# Supplementary material for: Deoxycholate induces proline provision from Escherichia coli to enhance Clostridioides difficile virulence
Source: ISME J. 2026 Sep 17;20(1):wrag249. doi: 10.1093/ismejo/wrag249 (PMC13634199; doi:10.1093/ismejo/wrag249)
Supplement: Supplementary file 1 [file supplementary_material_wrag249.zip › Figure S11.pdf]

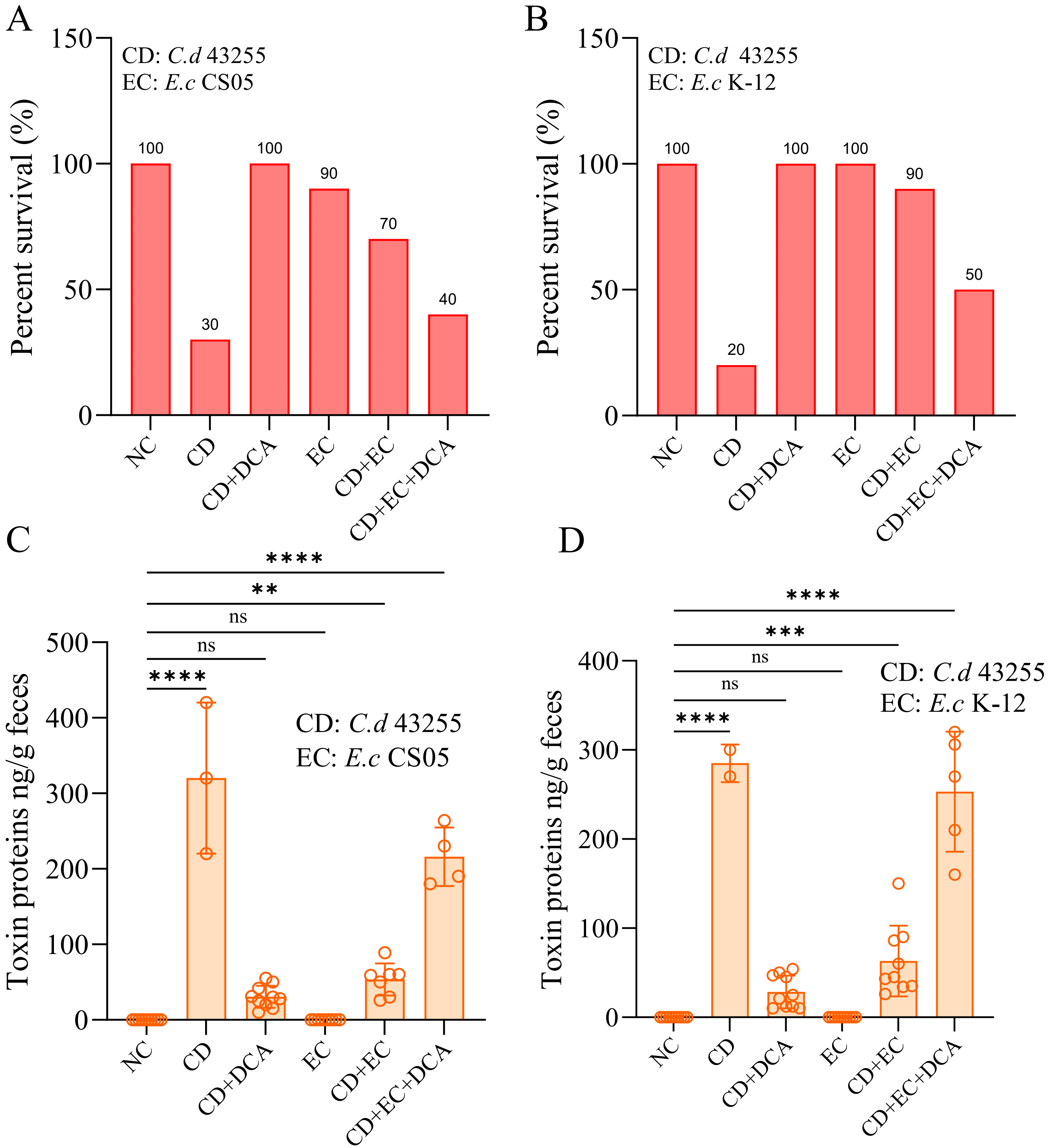

A
CD: C.d 43255
EC: E.c CS05
Percent survival (%)
150
100
50
0
100
30
100
90
70
40
NC
CD
CD+DCA
EC
CD+EC
CD+EC+DCA
B
CD: C.d 43255
EC: E.c K-12
Percent survival (%)
150
100
50
0
100
20
100
100
90
50
NC
CD
CD+DCA
EC
CD+EC
CD+EC+DCA
C
****
**
ns
ns
****
CD: C.d 43255
EC: E.c CS05
Toxin proteins ng/g feces
500
400
300
200
100
0
NC
CD
CD+DCA
EC
CD+EC
CD+EC+DCA
D
****
***
ns
ns
****
CD: C.d 43255
EC: E.c K-12
Toxin proteins ng/g feces
400
300
200
100
0
NC
CD
CD+DCA
EC
CD+EC
CD+EC+DCA
