# Supplementary material for: Deoxycholate induces proline provision from Escherichia coli to enhance Clostridioides difficile virulence
Source: ISME J. 2026 Sep 17;20(1):wrag249. doi: 10.1093/ismejo/wrag249 (PMC13634199; doi:10.1093/ismejo/wrag249)
Supplement: Supplementary file 1 [file supplementary_material_wrag249.zip › Supplementary_material.docx]

**Supplementary Data and Methods for:** **Deoxycholate induces proline provision from *Escherichia coli* to enhance** ***Clostridioides difficile* virulence**

Jingpeng Yang ^1, 2*^, Xilong Deng ^1, 2^, Xiaoya Sheng ^1, 2^, Yanan Li ^1, 2*^, Xuna Tang ^1, 3*^

^1^ Affiliated Hospital of Medical School, Nanjing Stomatological Hospital, Nanjing University Joint Research Center of Nanjing Normal University and Nanjing Stomatological Hospital, Nanjing 210008, China.

^2^ State Key Laboratory of Microbial Technology, Nanjing Normal University, 2 Xuelin Road, Qixia District, Nanjing, Jiangsu 210033, China.

^3^ Affiliated Hospital of Medical School, Nanjing Stomatological Hospital, Nanjing University, Nanjing, China.

***Corresponding author,**

Jingpeng Yang e-mail: [yang_jp008@163.com](mailto:yang_jp008@163.com) ORCID iD: 0000-0002-0802-2891

Yanan Li	e-mail: [liyanan@njnu.edu.cn](mailto:liyanan@njnu.edu.cn)

Xuna Tang	e-mail: [xunatang@126.com](mailto:xunatang@126.com)

**Detailed methods**

**Ecological interplay between *C. difficile* and *E. coli* in DCA-depleted environments**

Bacterial suspensions of *C. difficile* (CD) and *E. coli* (EC), each at an initial concentration of 3×10⁷ CFU/mL, were centrifuged at 12,000×g for 20 min. Following centrifugation, the resulting cell pellets and supernatants were harvested separately. The cell pellets were resuspended in fresh BHI broth to their original concentration (3×10⁷ CFU/mL), yielding the CD-cell and EC-cell suspensions. Concurrently, the supernatants were sterilized by filtration through a 0.22 μm membrane filter and subsequently mixed with fresh BHI broth at a 1:5 (v/v) ratio to prepare the cell-free culture supernatants (CFCS), designated as CD-CFCS and EC-CFCS.

To evaluate the effect of EC-CFCS on the growth of CD, 180 μL of EC-CFCS was mixed with 20 μL of CD-cell suspension (3×10⁷ CFU/mL) in each well of a 96-well microplate. The plate was incubated anaerobically at 37°C for 24 h. Bacterial growth was subsequently assessed by measuring the optical density at 630 nm using a microplate reader. For the negative control, EC-CFCS was replaced with an equal volume of fresh BHI broth. A reciprocal experiment was performed to assess the effect of CD-CFCS on EC growth by mixing 20 μL of EC-cell suspension with 180 μL of CD-CFCS, with BHI broth serving as the control. The impact of EC-CFCS on CD biofilm formation was determined using a crystal violet staining assay. Briefly, 100 μL of CD-cell suspension was mixed with 100 μL of EC-CFCS and inoculated into a 96-well plate. After a 48 h anaerobic incubation at 37°C, the supernatant was discarded, and the wells were gently washed twice with sterile PBS. The plates were then air-dried at 37°C for 15 min. Biofilms were stained by adding 200 μL of 0.2% (w/v) crystal violet solution to each well and incubating at 37°C for 30 min. Excess stain was removed by washing the wells twice with sterile PBS, followed by another 15 min drying period. The bound dye was solubilized with 200 μL of anhydrous ethanol, and the absorbance was measured at 570 nm to quantify biofilm. Wells containing only BHI broth were included as blank controls to correct for background absorbance. The effect of CD-CFCS on EC biofilm formation was evaluated using the same procedure. To quantify CD toxin levels, 20 μL aliquots were collected from the growth assays (CD+EC-CFCS) after the 24 h incubation. The samples were centrifuged at 12,000×g to pellet the cells, and the resulting supernatants were collected. The concentrations of toxins A and B were immediately quantified using the *C. difficile* TOXA/B II™ kit (TechLab, Blacksburg, VA, USA) according to the manufacturer’s instructions.

**Dose-dependent chemotaxis and aggregation of *C. difficile* in response to DCA**

CD cells were first pre-cultured in 2 mL of BHI broth containing various concentrations of DCA for 8 hours. Following this adaptation, the cells were harvested and fluorescently labeled by incubation with 10 μM Cell-Tracker Orange CMTMR (Shanghai Maokang Biotechnology Co., Ltd.) for 1 hour at 37°C under anaerobic conditions. The labeled cells were then washed twice and resuspended in chemotaxis buffer (10 mM potassium phosphate, 0.1 mM EDTA, pH 7.0). The cell suspension was adjusted to an optical density at 630 nm of 1.0. The chemotaxis assay was performed using a capillary method. Aliquots (200 μL) of the labeled CD suspension were dispensed into the wells of a 96-well plate. Glass capillaries (1 μL) were filled with one of the following chemoattractant solutions: chemotaxis buffer alone (negative control), buffer containing unlabeled EC cells, or buffer supplemented with 15 mM ethanolamine. Each capillary was then immersed into a well containing the CD suspension. The plate was incubated anaerobically at 37°C for 1 hour to allow for bacterial migration. After incubation, the contents of each capillary were carefully expelled, collected by centrifugation, and transferred to a new 96-well plate containing 200 μL of fresh LB medium. The fluorescence intensity of each well was measured using a microplate reader. The chemotactic response was quantified as a Chemotactic Index (CI), calculated using the following formula: CI (100%) = Fluorescence value of capillary centrifuge solution / Fluorescence value of initial solution×100%.

CD and EC cells, previously treated with varying concentrations of DCA, were harvested. Following two washes with sterile phosphate-buffered saline (PBS), the cells were resuspended in anaerobic aggregation buffer (150 mM NaCl, 1 mM Tris-HCl, 0.1 mM CaCl₂, 0.1 mM MgCl₂; pH 7.0). Both suspensions were adjusted to a final optical density at 630 nm of 1.0. To initiate the assay, 0.5 mL of the CD suspension was combined with 0.5 mL of the EC suspension in a cuvette. The mixture was vortexed for 10 seconds, and the initial optical density at 600 nm was immediately recorded (t = 0 h). The cuvette was then incubated at 37°C under anaerobic conditions for 1 hour. After incubation, the final 600 nm was measured (t = 1 h). The extent of bacterial aggregation was quantified as an Aggregation Index (AI), calculated using the following formula: AI = 100% – [(OD_600nm_ at 0 h – OD_600nm_ at 1 h) × 100%].

**Quantification of biofilm thickness and viable cell density via FISH-CLSM**

Based on preliminary DCA gradient experiments, three concentrations were selected for subsequent analysis: low (0.03125 mM; CEL), medium (0.125 mM; CEM), and high (1 mM; CEH). Biofilms were formed on sterile round coverslips placed at the bottom of 12-well plates. CD and EC cultures were adjusted to a final concentration of 3×10⁷ CFU/mL. For monocultures, 100 μL of a single strain was added to 2900 μL of BHI broth. For co-cultures, 100 μL of CD and 100 μL of EC were added to 2800 μL of BHI medium, either with or without the specified DCA concentrations. All plates were incubated anaerobically at 37°C for 48 hours to facilitate biofilm development. Following incubation, the biofilm-coated coverslips were gently washed with phosphate-buffered saline (PBS) to remove planktonic cells. For the analysis of total viable biomass, biofilms were stained with SYTO Green (Kage Bio, Nanjing, China), diluted 1: 2000 in fresh BHI broth, and incubated in the dark at room temperature for 1 hour. After a final PBS wash, the coverslips were mounted biofilm-side down onto microscope slides. The biofilms were then visualized using a confocal laser scanning microscope (CLSM; AX Confocal Microscope System, Nikon, Japan) equipped with a 60× oil immersion objective. Z-stack images were acquired with excitation/emission wavelengths set at 500/530 nm. In parallel, biofilms from duplicate samples were harvested by scraping into PBS, sonicated briefly to disperse aggregates, and serially diluted. The dilutions were then plated onto BHI agar plates. After incubation, viable cell counts of CD and EC were determined and distinguished based on their distinct colony morphologies.

To visualize the spatial distribution of each microbe within the biofilm, FISH was performed. Hybridization was conducted by mixing 100 ng of each species-specific probe with 20 μL of hybridization buffer (6× SSC, 5× Denhardt’s solution, 0.5% SDS, 100 μg/mL salmon sperm DNA, 50% formamide). The mixture was applied to the biofilm samples and incubated at 46°C in the dark for 5–6 h. Subsequently, samples were washed twice with washing buffer (20 mM Tris-HCl, 5 mM EDTA, 159 mM NaCl, 0.01% (w/v) SDS, pH 7.5) at 48°C for 20 min to remove excess probe. The samples were then imaged using the CLSM system. The FISH probes used in this study (Sangon Biotech, Shanghai, China) are as follows: CD-Specific probes, 5'-CATCCTGTACTGGCTCAC-3', 5'-Cy3-labeled; Ex/Em=550/570 nm [1]. EC-Specific probes, 5'-CTTTACTCCCTTCCTCCC-3', 5' labeled with Cy5, Ex/Em=640/670 nm [2].

**Modulation of *C. difficile* morphology and motility by DCA**

To investigate the dose-dependent effects of DCA on CD, three concentrations were selected: low (0.03125 mM), medium (0.125 mM), and high (1 mM). Experiments were conducted in both monoculture and co-culture with EC. Co-cultures were established using Transwell chambers with a 0.22 μm pore size to allow for the exchange of soluble metabolites whereas preventing direct cell contact. The experimental groups were defined as follows: Monoculture: CD-Control (no DCA), CD-Low, CD-Medium, CD-High. Co-culture: CE-Control (CD+EC, no DCA), CE-Low, CE-Medium, CE-High. All cultures were incubated anaerobically at 37°C for 48 hours. Following incubation, bacterial cells from each group were collected and subjected to Gram staining. Morphological changes were observed and documented using an optical microscope.

To assess motility, CD cells from the different treatment groups were cultured in fresh BHI broth and adjusted to an optical density at 630 nm of 0.5. Swimming Motility: A 5 μL aliquot of the standardized suspension was stab-inoculated into the center of 1/2 BHI plates containing 0.3% agar. The plates were incubated anaerobically at 37°C, and the diameter of the swimming zone was measured daily for 5 consecutive days.

Swarming Motility: A 5 μL spot of the standardized suspension was inoculated onto the surface of a freshly prepared (2-hour-old) 1/2 BHI plate containing 0.3% agar. The plates were incubated under the same conditions, and the swarming diameter was measured daily for 5 days. All assays were performed with three independent biological replicates.

**RNA-Sequencing (RNA-Seq) analysis**

To model the interaction between CD and EC, the transwell chambers (6.5 mm diameter inserts, 0.4 μm pore size, polycarbonate membrane, Corning, USA) were employed. CD and EC were inoculated at an equal density of 3×10⁷ CFU/mL into the inner and outer chambers of a 0.4 μm transwell system, respectively. The chambers were pre-incubated for 2 hours at 37°C under anaerobic conditions to allow for initial metabolic interaction. Following the pre-incubation, DCA was added to the co-cultures to establish the experimental groups: high (CEH, 1 mM), medium (CEM, 0.125 mM), and low (CEL, 0.03125 mM) concentrations. A DCA-free co-culture (CE) served as the control. All samples were then incubated for an additional 16 hours at 37°C under anaerobic conditions. After incubation, the cultures were processed for downstream analysis. The CD cells from the inner chamber were harvested by centrifugation to obtain cell pellets, which were designated for transcriptome sequencing. Simultaneously, the cell-free culture supernatant (CFCS) from the outer well was collected by filtration through a 0.22 μm sterile membrane. This CFCS was subsequently used for targeted or untargeted metabolomics analysis.

Total RNA was extracted from CD cells from each treatment group using the RNA-prep Pure Kit (TianGen Biotech, Beijing, China). The integrity, purity, and concentration of the extracted RNA were assessed using agarose gel electrophoresis, a NanoDrop 6090 spectrophotometer (Thermo Fisher Scientific, USA), and an Agilent 2100 Bioanalyzer (Agilent Technologies, Santa Clara, CA, USA). Ribosomal RNA (rRNA) was depleted from the total RNA using the Ribo-Zero rRNA Removal Kit (Bacteria). First-strand cDNA was synthesized from the enriched mRNA using random primers and SuperScript II Reverse Transcriptase (Invitrogen, Carlsbad, CA, USA). During second-strand synthesis, dUTP was incorporated instead of dTTP to facilitate strand specificity. The resulting double-stranded cDNA was then subjected to end-repair, A-tailing, and adapter ligation. The adapter-ligated fragments were purified, PCR-amplified, and quantified using the Quant-iT PicoGreen dsDNA Assay Kit (Invitrogen) on a Quantifluor-ST fluorometer (Promega). The finalized cDNA libraries were sequenced on an Illumina HiSeq platform using sequencing-by-synthesis (SBS) technology. The experiment included at least three independent biological replicates per treatment group. Raw sequencing data in FASTQ format underwent quality control to remove low-quality reads and adapter sequences. The resulting high-quality reads were then aligned to the reference genome of *C. difficile* ATCC 43255 (NCBI Accession: GCF_000155025.1) using Bowtie2, generating sequence alignment maps for the CE, CEL, CEM, and CEH groups for downstream differential expression analysis.

**Untargeted metabolomic profiling**

Metabolites were extracted from fresh samples (bacterial suspensions or mouse feces). An aliquot of each sample was mixed with an ice-cold solvent mixture (methanol: acetonitrile: water, 2:2:1, v/v) and vortexed thoroughly. The mixture was then sonicated for 30 min at -20°C, followed by a 10-minute incubation on ice. After centrifugation at 14,000×g for 20 min at 4°C, the resulting supernatant was collected and dried under vacuum. The dried residue was reconstituted in 100 μL of a 1:1 (v/v) acetonitrile-water solution, vortexed, and centrifuged. The final supernatant was transferred to autosampler vials for LC-MS analysis.

Chromatographic separation was performed on a Vanquish UHPLC system (Thermo Fisher Scientific) equipped with a HILIC column. The column temperature was maintained at 25°C with a flow rate of 0.3 mL/min, and the injection volume was 2 μL. The mobile phase consisted of (A) water with 25 mM ammonium acetate and 25 mM ammonium hydroxide, and (B) acetonitrile. A gradient elution program was applied as follows: 98% B (0–1.5 min), a linear decrease to 2% B (1.5–12 min), hold at 2% B (12–14 min), a linear increase to 98% B (14–14.1 min), and re-equilibration at 98% B (14.1–17 min). Samples were maintained at 4°C in the autosampler. Mass spectrometry detection was carried out on a Q Exactive series mass spectrometer (Thermo Fisher Scientific) equipped with an electrospray ionization (ESI) source, operating in both positive and negative ion modes. The ESI source parameters were set as follows: sheath gas flow, 60; auxiliary gas flow, 60; capillary temperature, 600°C; and spray voltage, ±5500 V. For MS1 full scans, the m/z range was 80–1200 Da with a resolution of 60,000. For data-dependent MS/MS acquisition, the top N most intense ions were fragmented with a resolution of 30,000, and dynamic exclusion was set to 4 s. To ensure data quality, a pooled quality control (QC) sample was prepared by mixing equal aliquots of all samples. The injection sequence was randomized, and the QC sample was injected at regular intervals to monitor instrument stability and data reliability.

Raw data files were converted to the ‘.mzXML’ format using ProteoWizard. Peak detection, retention time correction, and peak alignment were performed using the XCMS software. The resulting data matrix was then subjected to metabolite annotation and preprocessing. Following a rigorous quality assessment of the experimental data, the processed dataset was used for subsequent statistical analysis.

**Targeted metabolomic profiling**

A series of amino acid standard solutions was prepared at concentrations of 10, 20, 50, 100, 200, 500, 1000, 2000, 5000, 10000, and 20000 ng/mL. Calibration curves for each amino acid were constructed by plotting the peak area ratio (analyte/internal standard) against the corresponding concentration ratio (analyte concentration/internal standard concentration). The linear regression equation derived from each curve was used for subsequent quantification. For bacterial suspensions, 50 μL of the sample was mixed with 250 μL of a 20% acetonitrile-methanol extraction solution. For mouse fecal samples, 50 mg (± 2.5 mg) of the sample was homogenized in 500 μL of pre-chilled (-20°C) 70% methanol-water and 400 μL of chloroform. All mixtures were vortexed for 3 min and centrifuged at 12,000 rpm and 4°C for 10 min. A 250 μL aliquot of the supernatant was transferred to a new tube and stored at -20°C for 30 min, followed by a second centrifugation under the same conditions. Finally, 200 μL of the clarified supernatant was transferred to a sample vial for UPLC-MS/MS analysis. The remaining extract was stored at -20°C.

Chromatographic separation was performed on an ExionLC™ AD UPLC system (SCIEX) equipped with an ACQUITY BEH Amide column (1.7 μm, 100 mm × 2.1 mm i.d.). The column temperature was maintained at 40°C with a flow rate of 0.4 mL/min, and the injection volume was 2 μL. The mobile phase consisted of (A) ultrapure water and (B) acetonitrile, both containing 2 mM ammonium acetate and 0.04% formic acid. The gradient elution program was as follows: 0–1.2 min, 10% A; 1.2–9 min, linear increase to 40% A; 9–10 min, linear increase to 60% A; 10–11 min, hold at 60% A; 11.01–15 min, return to 10% A for re-equilibration. Mass spectrometric detection was conducted on a QTRAP® 6500+ triple quadrupole instrument (SCIEX) equipped with an electrospray ionization (ESI) source, operating in both positive and negative ion modes. The source parameters were set as follows: ion spray voltage, +5500 V (positive) / -4500 V (negative); source temperature, 550°C; curtain gas (CUR), 35 psi. Each amino acid was quantified using Multiple Reaction Monitoring (MRM) with optimized declustering potential (DP) and collision energy (CE). Data were acquired using Analyst 1.6.3 software. To ensure analytical quality, a pooled quality control (QC) sample was prepared and injected after every 10 analytical samples. Instrument stability was assessed by overlaying the total ion current (TIC) profiles of the QC injections.

The raw data were processed using MultiQuant 3.0.3 software. Chromatographic peaks were identified by referencing the retention times of the standards and subsequently integrated. The peak area ratios were substituted into the corresponding linear regression equations to calculate the concentration of each amino acid in the extracted solution (c, ng/mL). The final concentration in the original sample was determined using the following formula to account for the dilution factor: Concentration of each substance in the sample (ng/mL) = c×V1/1000/V2. Explanation of variables: c: Concentration value (ng/mL) obtained by substituting the integrated peak area in the sample into the standard curve. V1: Volume of extraction solution used (μL). V2: Volume of sample transferred (mL).

**Murine model of CDI**

**Experiment 1:** Six-week-old male C57BL/6J mice were purchased from Shanghai Slake Experimental Animal Co., Ltd. (Shanghai, China) and acclimatized for one week before experimentation. All mice were housed individually under identical conditions and confirmed to be CD toxin-negative before the study. All animal procedures were approved by the Animal Ethics Committee of Nanjing Normal University (IACUC-2603044). A CDI model was established by adapting previously reported protocols [3-5]. Mice were randomly assigned (n=10 per group) to one of eight groups: two control groups (NCn and NCa), two single-bacterium infection groups (CD and EC), one co-infection group (CE), and three co-infection groups with DCA treatment (CEL, CEM, CEH). Except for the NCn group, all mice received a 7-day course of antibiotics (1 mg/mL cefoperazone) in their drinking water to disrupt the gut microbiota, followed by a 2-day recovery period on normal water. Subsequently, the infection phase was initiated via oral gavage. Mice in the CD group were inoculated with 3×10⁸ CFU of *C. difficile* ATCC 43255. Mice in the EC group received 3×10⁸ CFU of *E. coli* ATCC 25922. Mice in the co-infection groups (CE, CEL, CEM, CEH) were inoculated with a suspension containing both 3×10⁸ CFU of *C. difficile* and 3×10⁸ CFU of *E. coli*. For the DCA treatment groups (CEL, CEM, and CEH), mice were subjected to oral gavage with escalating concentrations of DCA (0.03125 mM, 0.125 mM, and 1 mM, respectively) beginning 12 hours post-infection, and maintained for the duration of the study (until CDI symptoms disappeared). Throughout the experiment, mice were monitored daily for body weight, survival, and clinical signs of disease. Disease severity was scored on a scale from 0 (normal) to 20 (near death) [6]. Humane endpoints were strictly observed; mice were euthanized when they became moribund or exhibited weight loss exceeding 20% of their baseline body weight. Fecal samples were collected at four key time points: T1 (pre-antibiotic), T2 (pre-infection), T3 (one day post-infection), and T4 (study endpoint). These samples were used for quantification of viable *C. difficile* and *E. coli*, measurement of CD toxin and fecal lipocalin-2 (Lcn2), and for microbiome and metabolome analyses.

**Experiment 2:** This experiment aimed to evaluate the impact of continuous administration of varying DCA concentrations on the levels of key bile acid synthesis enzymes in the livers of uninfected mice. Mice were randomly assigned to four groups (n=6 per group): a control group (NC) and low-, medium-, and high-dose DCA intervention groups (L-DCA, M-DCA, and H-DCA, respectively). The concentration and duration of the DCA intervention were consistent with those of Experiment 1.

**Experiment 3:** Building upon Experiment 1, two additional mono-infection groups were established: the CD+DCA group (n=10) and the EC+DCA group (n=10). Throughout the infection period, both groups received continuous oral gavage of high-dose DCA (1 mM). The DCA administration schedule was consistent with that of Experiment 1.

**Experiment 4:** The methodology for Experiment 4 was identical to that of Experiment 1, with the key substitution of *C. difficile* ATCC 43255 with *C. difficile* ATCC 9689. Mice were randomly assigned (n=10 per group) to six experimental groups: a non-infected control (NC), single-bacterium infections (CD, EC), a co-infection (CD+EC), and DCA-treated infections (CD+DCA, CD+EC+DCA). All groups received the same 7-day antibiotic pretreatment as in Experiment 1, followed by a 2-day washout period. Subsequently, mice were infected via oral gavage with 3×10⁸ CFU of the respective bacteria: CD (*C. difficile* ATCC 9689), EC (*E. coli* ATCC 25922), or CD+EC (both strains at 3×10⁸ CFU). The NC control group received antibiotic pretreatment but was gavaged with sterile vehicle and did not receive DCA. For the DCA-treated groups, mice in the CD+DCA and CD+EC+DCA groups were provided with drinking water containing 1 mM DCA, initiated 12 hours post-infection. All other experimental procedures, including clinical monitoring and sample collection, were performed as described in Experiment 1.

**Experiment 5:** The methodology for Experiment 5 was identical to Experiment 1, with the substitution of *C. difficile* ATCC 43255 with the wild-type *C. difficile* 630 strain and its isogenic mutant, 630ΔprdB (a *prdB* gene deletion strain). Mice were randomly assigned (n=10 per group) to ten experimental groups: a non-infected control (NC), three single-infection groups, two co-infection groups, and four DCA-treated infection groups. All groups, except NC, underwent the same 7-day antibiotic pretreatment followed by a 2-day washout period. Subsequently, mice were infected via oral gavage with 3×10⁸ CFU of the specified bacteria. The single-infection groups received either *C. difficile* 630 (CD), *C. difficile* 630ΔprdB (ΔprdB), or *E. coli* ATCC 25922 (EC). The co-infection groups (CD+EC, ΔprdB+EC) received a suspension containing both 3×10⁸ CFU of the respective *C. difficile* strain and 3×10⁸ CFU of *E. coli*. The NC control group received antibiotic pretreatment but was gavaged with sterile vehicle. For the DCA-treated groups (CD+DCA, ΔprdB+DCA, CD+EC+DCA, ΔprdB+EC+DCA), drinking water containing 1 mM DCA was administered 12 hours post-infection and continued for the duration of the study. All other experimental procedures, including clinical monitoring and sample collection, were performed as described in Experiment 1.

**Experiment 6:** The methodology for Experiment 6 was identical to Experiment 1, with the substitution of *E. coli* ATCC 25922 with the *E. c* CS05 or *E. c* K-12 strain. This phase of the experiment aimed to determine whether co-infection with different *E. coli* strains and *C. difficile* ATCC 43255 produces a consistent trend in mortality. For these groups, DCA was administered at a concentration of 1 mM. At the study endpoint, the final survival rates of the mice and fecal *C. difficile* toxin levels were evaluated.

**Determination of the levels of some key bile acid production enzymes in the liver**

Hepatic tissues were harvested from mice following seven consecutive days of intragastric DCA administration. With slight modifications based on the method described by Chen et al. [26], mouse livers were harvested, weighed, and immediately snap-frozen in liquid nitrogen for subsequent analysis. Hepatic microsomes were prepared by differential ultracentrifugation. The protein expression levels of CYP7A1, CYP8B1, CYP27A1, and CYP7B1 were quantified using commercial ELISA kits: Mouse CYP7A1 ELISA Kit (ELK3901, ELK Biotechnology), Mouse CYP8B1 ELISA Kit (FT-P9S2565X, Fantai Bio), Mouse CYP27A1 ELISA Kit (FT-P9S2564X, Fantai Bio), and Mouse CYP7B1 ELISA Kit (EF014613, LSM Bio). All samples were prepared and diluted in strict accordance with the manufacturers’ instructions prior to the assay.

**Histopathological evaluation and clinical disease assessment**

Cecal and colonic tissues were harvested and fixed in 10% neutral-buffered formalin. After paraffin embedding, 5 μm sections were prepared and stained with Hematoxylin and Eosin (H&E). Histopathological scoring was performed by a blinded observer according to a previously established system [23]. A total score (0-12) was derived by summing the individual scores (0-4) for three parameters: epithelial damage, inflammatory cell infiltration, and submucosal edema. The detailed scoring criteria were as follows. Epithelial damage was scored from 0 (no changes) to 4 (marked pseudomembrane or ulceration). Inflammatory cell infiltration was graded from 0 (no inflammation) to 4 (severe confluent infiltrate with abscess formation). Submucosal edema was assessed from 0 (no edema) to 4 (severe, diffuse submucosal dilation). The complete descriptions for each score level were based on the defined criteria, which assessed the severity and extent of tissue damage, immune cell presence, and fluid accumulation.

**Fecal microbiota profiling by 16S rRNA gene sequencing**

Fecal genomic DNA was extracted using the OMEGA Soil DNA Kit (D5625-01, Omega Bio-Tek, Norcross, GA, USA) according to the manufacturer’s protocol. The concentration and purity of DNA were measured using a NanoDrop ND-1000 spectrophotometer (Thermo Fisher Scientific, Waltham, MA, USA), whereas its integrity was verified by 1.2% agarose gel electrophoresis. Purified DNA was stored at -20°C until further processing. The V3-V4 region of the bacterial 16S rRNA gene was amplified using the forward primer 338F (5’-ACTCCTACGGGAGGCAGCA-3’) and the reverse primer 806R (5’-GGACTACHVGGGTWTCTAAT-3’). PCR was performed in a 25 μL reaction mixture containing 5 μL of 5× buffer, 0.25 μL of Fast Pfu DNA Polymerase (5 U/μL), 2 μL of dNTPs (2.5 mM), 1 μL of each primer (10 μM), 1 μL of template DNA, and 14.75 μL of nuclease-free water. The thermal cycling conditions were as follows: initial denaturation at 98°C for 5 min; 25 cycles of denaturation at 98°C for 30 s, annealing at 53°C for 30 s, and extension at 72°C for 45 s; and a final extension at 72°C for 5 min. A no-template control was included in each PCR batch to monitor for potential contamination. The resulting amplicons were purified using Vazyme VAHTSTM DNA Clean Beads (Vazyme, Nanjing, China) and quantified with the Quant-iT PicoGreen dsDNA Assay Kit (Invitrogen, Carlsbad, CA, USA). Purified amplicons were then pooled in equimolar concentrations and subjected to paired-end sequencing on an Illumina MiSeq platform using the MiSeq Reagent Kit V3 (Illumina, San Diego, CA, USA). Sequencing data were processed and analyzed using the QIIME 2 platform (version 2019.4). Raw reads were first demultiplexed, and primers were trimmed using the demux and cutadapt plugins, respectively. Subsequently, the DADA2 pipeline was employed for quality filtering, denoising, paired-end read merging, and chimera removal to generate amplicon sequence variants (ASVs). Representative sequences for each ASV were aligned using mafft, and a phylogenetic tree was constructed with fasttree2 for downstream phylogenetic diversity analyses.

**Fecal microbiome profiling by shotgun metagenomics**

Sequencing libraries were constructed from fecal genomic DNA using the Illumina TruSeq DNA PCR-Free Library Prep Kit (or Illumina TruSeq DNA Sample Prep Kit) according to the manufacturer’s protocol. Initially, genomic DNA was sheared into fragments of approximately 350-450 bp using a Bioruptor sonicator. The fragmented DNA was then subjected to end-repair and A-tailing, followed by ligation of indexed TruSeq adapters. Post-ligation, the libraries were purified using AMPure XP beads to remove adapter dimers and select for the desired fragment size. The adapter-ligated fragments were enriched with a minimal number of PCR cycles to mitigate amplification bias. The final libraries were quantified using a Qubit 4 Fluorometer and assessed for fragment size distribution and integrity using an Agilent 2100 Bioanalyzer. Individually indexed libraries were normalized to 10 nM and pooled in equimolar concentrations. The pooled library was then diluted to the optimal loading concentration and sequenced on an Illumina NovaSeq X Plus platform using a 2×150 bp paired-end configuration.

**Bioinformatics analysis**

**Transcriptome:** Gene expression levels were initially quantified as Fragments Per Kilobase of transcript per Million mapped reads (FPKM) using HTSeq (version 0.6.1). Differential expression analysis between experimental groups was subsequently performed using the DESeq package (version 1.18.0). To account for multiple hypothesis testing, p-values were adjusted using the Benjamini-Hochberg method to control the False Discovery Rate (FDR). Genes were considered significantly differentially expressed (DEGs) if they met the criteria of an adjusted p-value (FDR) < 0.05 and an absolute log₂ fold change (|log₂FC|) > 1. To visualize these results, volcano plots and hierarchical clustering heatmaps were generated using the ggplot2 and pheatmap packages in R, respectively. For functional annotation, DEGs were subjected to KEGG (Kyoto Encyclopedia of Genes and Genomes) pathway enrichment analysis. The significance of pathway enrichment was determined by three metrics: the Rich factor, the FDR, and the count of DEGs within the pathway. The Rich factor, defined as the ratio of DEGs to the total number of annotated genes in a given pathway, indicates the degree of enrichment. A lower FDR value signifies more statistically significant enrichment. The top 20 most significantly enriched pathways, ranked by the lowest FDR, were selected for visualization.

**Metabolome:** Metabolite identification was achieved by querying both proprietary (Piseno, Shanghai, China) and public databases, including the Human Metabolome Database (HMDB), METLIN, MassBank, and mzCloud. A multi-parameter matching strategy was employed, wherein metabolites were structurally characterized based on retention time, precise molecular weight (mass error < 10 ppm), and tandem mass spectrometry (MS/MS) fragmentation patterns. All identifications were rigorously validated and assigned a confidence level of 2 or higher, in accordance with the Metabolomics Standards Initiative (MSI). Subsequently, the identified metabolites were taxonomically classified. Unsupervised Principal Component Analysis (PCA) was initially performed to visualize the overall metabolic landscape and assess intrinsic variations among the samples. To identify metabolites contributing significantly to group discrimination, a supervised Orthogonal Partial Least Squares-Discriminant Analysis (OPLS-DA) model was constructed. Metabolites with a Variable Importance in Projection (VIP) score exceeding 1.0 and a p-value < 0.05 were defined as significantly differentially abundant. These key metabolites were then mapped to the Kyoto Encyclopedia of Genes and Genomes (KEGG) database for pathway enrichment analysis. This analysis integrated Over-Representation Analysis (ORA) to evaluate the statistical significance of metabolite enrichment within specific pathways, and pathway topology analysis to assess the relative impact and biological importance of these pathways in the context of the studied phenomenon.

**Microbiome:** Alpha diversity within samples was evaluated using the Shannon and Observed Species indices, with statistical significance of inter-group differences assessed via the Kruskal-Wallis test followed by Dunn’s post-hoc test. Beta diversity, representing compositional dissimilarity between samples, was calculated using Bray-Curtis and Jaccard dissimilarity metrics and visualized through non-metric multidimensional scaling (NMDS). To further investigate sample relationships, hierarchical clustering was performed using the Unweighted Pair-Group Method with Arithmetic Means (UPGMA), and the resulting dendrogram was visualized with the ggtree package in R. For the identification of differentially abundant taxa, two complementary approaches were employed. First, a random forest algorithm (R package randomForest) was used to pinpoint species whose abundance significantly contributed to group discrimination. Second, Linear Discriminant Analysis Effect Size (LEfSe) analysis was conducted across all taxonomic levels to identify robust biomarkers, which were then visualized in a cladogram illustrating their taxonomic distribution [7]. Functional relationships among samples were depicted using Circos plots generated with the circlize package. Finally, a Procrustes analysis was performed to test for concordance between microbial taxonomy and function. This method correlated the principal coordinates analysis (PCoA) ordinations of species composition and functional abundance profiles, and a permutation test (e.g., the procrustes function in vegan) was used to evaluate the statistical significance of their association, thereby determining if the two dimensions exhibited consistent patterns of variation.

**Multi-omics correlation analysis**: To investigate the overall relationship between the metabolome and microbiome, a two-pronged approach was employed. First, Bray-Curtis dissimilarity matrices were calculated separately for metabolomic and microbial abundance data using the vegan package in R. A Mantel test with 999 permutations was then performed using QIIME software to assess the correlation between these two dissimilarity matrices. Second, a Procrustes analysis was conducted on the principal coordinates analysis (PCoA) ordinations of both datasets using QIIME. This analysis evaluated the concordance between the metabolomic and microbial compositional profiles, with statistical significance determined via a permutation test (999 permutations) yielding an M² statistic and a corresponding p-value. Following the confirmation of a significant overall association, we sought to identify specific metabolite-microbe interactions. Spearman’s rank correlation coefficients were calculated between individual metabolite abundances and microbial taxonomic abundances using Mothur software. Based on the resulting correlation coefficients (rho) and their associated p-values, significant associations were selected and visualized in a heatmap generated in R.

**Reference**

1.	Semenyuk EG, Poroyko VA, Johnston PF *et al.* Analysis of bacterial communities during clostridium difficile infection in the mouse. *Infect Immun*. 2015;**83**:4383-91 <https://doi.org/10.1128/IAI.00145-15>

2.	Wu S, Ueno D, Inoue K *et al.* Direct viable count combined with fluorescence in situ hybridization (dvc-fish) for specific enumeration of viable escherichia coli in cow manure. *Microbes and Environments*. 2009;**24**:33-38 <https://doi.org/10.1264/jsme2.ME08543>

3.	Theriot CM, Koumpouras CC, Carlson PE *et al.* Cefoperazone-treated mice as an experimental platform to assess differential virulence ofclostridium difficilestrains. *Gut Microbes*. 2014;**2**:326-34 <https://doi.org/10.4161/gmic.19142>

4.	Yang J, Yang H. Evaluation of the therapeutic effect and dose–effect of bifidobacterium breve on the primary clostridioides difficile infected mice. *Applied Microbiology and Biotechnology*. 2021 <https://doi.org/10.1007/s00253-021-11668-2>

5.	Smith AB, Jenior ML, Keenan O *et al.* Enterococci enhance clostridioides difficile pathogenesis. *Nature*. 2022 <https://doi.org/10.1038/s41586-022-05438-x>

6.	De Wolfe TJ, Kates AE, Barko L *et al.* Modified mouse model of clostridioides difficile infection as a platform for probiotic efficacy studies. *Antimicrob Agents Chemother*. 2019;**63** <https://doi.org/10.1128/aac.00111-19>

7.	Segata N, Izard J, Waldron L *et al.* Metagenomic biomarker discovery and explanation. *Genome Biology*. 2011;**12**:R60

**Fig. S1 Viability and cellular morphology of *C. difficile* (CD) within symbiotic biofilms.** A. Quantification of viable CD and EC cells within symbiotic biofilms formed in the presence of DCA. B. Morphological changes in CD cells when co-cultured with EC under DCA exposure.

**Fig. S2 Transcriptomic and metabolomic profiling of *C. difficile* in co-culture with DCA.** A. Volcano plot of differentially expressed genes (DEGs). Red and blue dots represent significantly upregulated and downregulated genes, respectively; gray dots indicate genes with no significant change. B. Principal component analysis (PCA) of metabolites across different treatment groups. Each point represents an individual sample, color-coded by group, with the ellipse indicating the 95% confidence interval. C. Heatmap of the top 40 most abundant metabolites across treatment groups. Red and blue colors denote high and low abundance, respectively. D. Importance plot from Orthogonal Partial Least Squares-Discriminant Analysis (OPLS-DA). Metabolites are plotted by their Variable Importance in Projection (VIP) score (x-axis) against their false discovery rate (FDR)-corrected *P* value (log10 transformed, y-axis).

**Fig. S3 Effects of continuous intragastric administration of varying doses of DCA over 7 days on the hepatic expression levels of CYP7A1 (A), CYP7B1 (B), CYP8B1 (C), and CYP27A1 (D) in mice. (E) Survival rates of mice post-infection.** NC, normal control group; L-DCA, M-DCA, and H-DCA, low-, medium-, and high-dose DCA intervention groups, respectively; CD+DCA and EC+DCA, high-dose DCA intervention groups. For all panels, statistical significance was determined by Dunnett's test: ns, not significant (*P* > 0.05); **P* < 0.05; ***P* < 0.01; ****P* < 0.001; *****P* < 0.0001.

**Fig. S4 Animal experiments.** A. Levels of *C. difficile* toxin proteins and lipocalin-2 (Lcn2) in feces at different time points. B. Representative H&E-stained cecal tissue sections at the final time point (T4). Significant differences were analyzed using one-way ANOVA, followed by Tukey’s multiple comparisons test. Different lowercase letters indicate statistically significant differences between groups (*P* < 0.05).

**Fig. S5 Fecal microbiota composition analysis at time point T3.** A. Top 20 microbes by relative abundance at the family level. B. Alpha diversity indices across different groups. C. Non-metric multidimensional scaling (NMDS) plot based on the Bray-Curtis dissimilarity matrix. D. Hierarchical clustering analysis (UPGMA) of samples based on the Bray-Curtis distance, visualized with a heatmap of the top 15 most abundant bacteria. E. Heatmap of bacterial composition ranked by average abundance (top 20). F. Random forest analysis identifying the top 20 most important species-level taxa for group discrimination. Bar length indicates the mean decrease in accuracy (importance score). G. Circos plot illustrating the functional composition of the microbiome. The left semicircle shows the top 10 functional units, and the right semicircle shows the samples. Links indicate the contribution of each functional unit to the samples. H. Cladogram generated by Linear Discriminant Analysis Effect Size (LEfSe), identifying biomarkers from phylum to genus (from inner to outer circles). The color of each node represents the group in which the biomarker is most abundant.

**Fig. S6 Longitudinal analysis of fecal microbiota composition.** A. Relative abundance of the top 15 bacterial families across different time points. B. Alpha diversity indices over time. C. Non-metric multidimensional scaling (NMDS) plot based on the Bray-Curtis dissimilarity matrix for samples at various time points. D. Hierarchical clustering analysis (UPGMA) of samples based on the Bray-Curtis distance, visualized with a heatmap of the top 15 most abundant bacteria at the species level. E. Random forest analysis identifying the top 20 most important species-level taxa for discriminating between time points. Bar length indicates the importance score for each taxon. F. Procrustes analysis correlating species composition with functional composition. Each pair of connected points (solid and hollow circles) represents a single sample, with line length indicating the degree of concordance between its species and functional profiles.

**Fig. S7 Fecal metabolomic profiles of mice from different treatment groups at T3.** A. Functional classification of detected metabolites based on their chemical categories. B. Principal component analysis (PCA) of metabolites, illustrating inter-sample variability and clustering by treatment group.

**Fig. S8 Quantification of specific fecal metabolites.** A. Relative abundance of primary bile acids. B. Relative abundance of secondary bile acids. C. Relative abundance of key amino acids in the Stickland pathway. Significant differences were analyzed using one-way ANOVA, followed by Tukey’s multiple comparisons test. Different lowercase letters indicate statistically significant differences between groups (*P* < 0.05).

**Fig. S9 Validation of D-proline utilization and toxin enhancement across different *C. difficile* strains.** A. Utilization of D-proline by the wild-type *C. d* 630 strain and its isogenic *prdB* knockout mutant (ΔprdB) with or without 1 mM DCA. B. Toxin production by various *C. difficile* clinical isolates when co-cultured with *E. coli* (EC) in the presence or absence of 1 mM DCA. For all panels, statistical significance was determined by a two-tailed Student’s t-test: ns, not significant (*P* > 0.05); **P* < 0.05; ***P* < 0.01; ****P* < 0.001; *****P* < 0.0001.

**Fig. S10 Validation of the *prdB*-mediated mechanism in a murine model using additional *C. difficile* strains.** Survival rates of mice following co-infection with *E. coli* 25922 and *C. difficile* 9689 (A) or the *C. difficile* 630 wild-type (WT) and ΔprdB mutant (B), under DCA treatment. C. D-proline content in feces of mice co-infected with CD9689 and EC25922 at the T3 time point (post-infection). D. D-proline content in feces of mice co-infected with CD630 (CD630ΔprdB) and EC25922 at the T3 time point (post-infection). E. Toxin protein content in feces of mice co-infected with CD9689 and EC25922 at T3 (post-infection). F. Toxin protein content in feces of mice co-infected with CD630 (CD630ΔprdB) and EC25922 at T3 (post-infection). For all panels, statistical significance was determined by Dunnett's test: ns, not significant (*P* > 0.05); **P* < 0.05; ***P* < 0.01; ****P* < 0.001; *****P* < 0.0001.

**Fig. S11 Evaluation of murine models co-infected with CD ATCC 43255 and different *Escherichia coli* strains.** Survival rates of mice co-infected with CD 43255 and (A) EC CS05 or (B) EC K-12. (C, D) Fecal toxin protein levels at T3 (post-infection) in mice co-infected with CD 43255 and EC CS05 or EC K-12. For all panels, statistical significance was determined by Dunnett's test: ns, not significant (*P* > 0.05); **P* < 0.05; ***P* < 0.01; ****P* < 0.001; *****P* < 0.0001.

**Table S1. Primers for real-time qPCR**

| **Gene** | **Forward primer sequence** | **Reverse primer sequence** |
| --- | --- | --- |
| *CD-*16S rRNA | AGAGTTTGATCCTGGCTCAG | GGTTACCTTGTTACGACTT |
| *prdB* | TGCCATAGAACTGCCGTGAT | TCACCAGCATTAGCACCCATT |
| *prdF* | GGTGGTGGTATACTGCTGGT | CAGCACCTTCAGTAACATCC |
| *tcdA* | CAGTCGGATTGCAAGTAATTGACAAT | AGTAGTATCTACTACCATTAACAGTCTGC |
| *tcdB* | TACAAACAGGTGTATTTAGTACAGAAGATGGA | CACCTATTTGATTTAGMCCTTTAAAAGC |
| *EC-16S rRNA* | AGAGTTTGATCCTGGCTCAG | GGTTACCTTGTTACGACTT |
| *proB* | CTGAACCGTGCCCATATCGT | AGTTGTTTCGAGGCGATGGT |
| *putP* | TGTCCATGCGCGTCGTATTA | GGCGTTCTGATTTACCGCAC |
