# Supplementary material for: Deoxycholate induces proline provision from Escherichia coli to enhance Clostridioides difficile virulence
Source: ISME J. 2026 Sep 17;20(1):wrag249. doi: 10.1093/ismejo/wrag249 (PMC13634199; doi:10.1093/ismejo/wrag249)
Supplement: Supplementary file 1 [file supplementary_material_wrag249.zip › Figure S1.pdf]

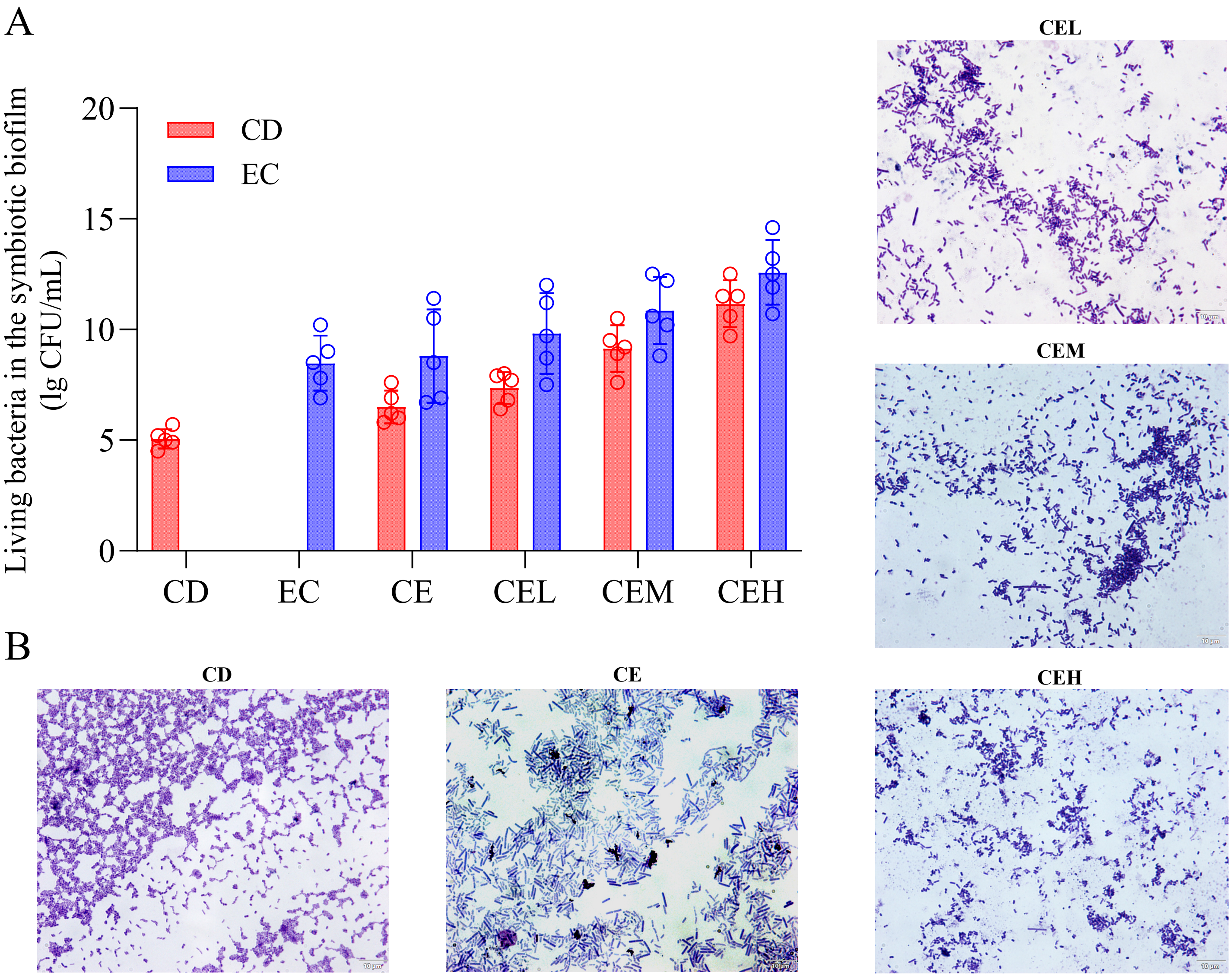
A
B
Living bacteria in the symbiotic biofilm (lg CFU/mL)
20
15
10
5
0
CD
EC
CD
EC
CE
CEL
CEM
CEH
CEL
CEM
CEH
CD
CE
10 μm
