# Supplementary material for: Deoxycholate induces proline provision from Escherichia coli to enhance Clostridioides difficile virulence
Source: ISME J. 2026 Sep 17;20(1):wrag249. doi: 10.1093/ismejo/wrag249 (PMC13634199; doi:10.1093/ismejo/wrag249)
Supplement: Supplementary file 1 [file supplementary_material_wrag249.zip › Figure S2.pdf]

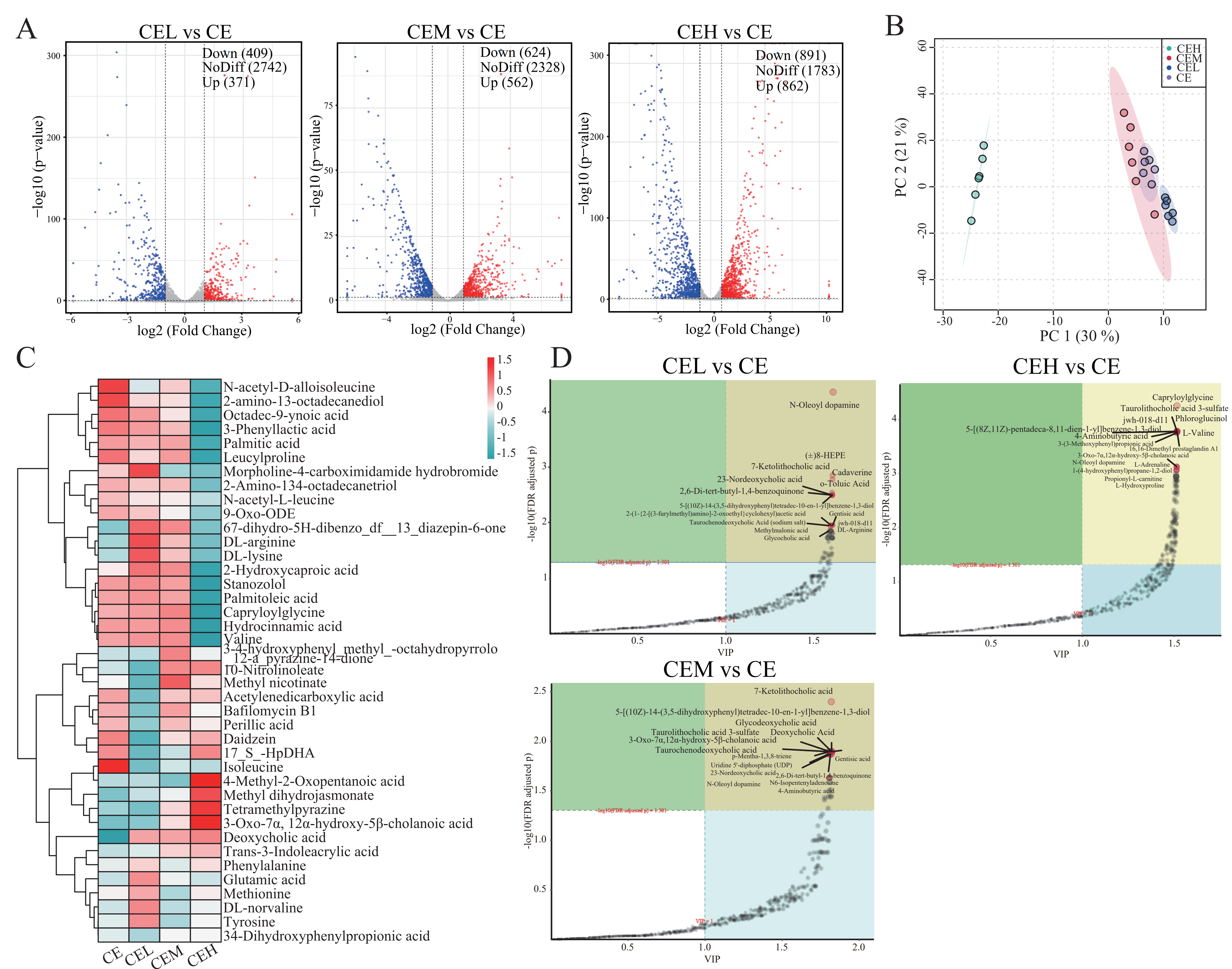
A
CEL vs CE
Down (409)
NoDiff (2742)
Up (371)
−log10 (p−value)
log2 (Fold Change)
CEM vs CE
Down (624)
NoDiff (2328)
Up (562)
CEH vs CE
Down (891)
NoDiff (1783)
Up (862)
B
CEH
CEM
CEL
CE
PC 2 (21 %)
PC 1 (30 %)
C
N-acetyl-D-alloisoleucine
2-amino-13-octadecanediol
Octadec-9-ynoic acid
3-Phenyllactic acid
Palmitic acid
Leucylproline
Morpholine-4-carboximidamide hydrobromide
2-Amino-134-octadecanetriol
N-acetyl-L-leucine
9-Oxo-ODE
67-dihydro-5H-dibenzo_df__13_diazepin-6-one
DL-arginine
DL-lysine
2-Hydroxycaproic acid
Stanozolol
Palmitoleic acid
Capryloylglycine
Hydrocinnamic acid
Valine
3-4-hydroxyphenyl_methyl_-octahydropyrrolo_12-a_pyrazine-14-dione
10-Nitrolinoleate
Methyl nicotinate
Acetylenedicarboxylic acid
Bafilomycin B1
Perillic acid
Daidzein
17_S_-HpDHA
Isoleucine
4-Methyl-2-Oxopentanoic acid
Methyl dihydrojasmonate
Tetramethylpyrazine
3-Oxo-7α, 12α-hydroxy-5β-cholanoic acid
Deoxycholic acid
Trans-3-Indoleacrylic acid
Phenylalanine
Glutamic acid
Methionine
DL-norvaline
Tyrosine
34-Dihydroxyphenylpropionic acid
CE
CEL
CEM
CEH
D
CEL vs CE
N-Oleoyl dopamine
(±)8-HEPE
7-Ketolithocholic acid
Cadaverine
23-Nordeoxycholic acid
o-Toluic Acid
2,6-Di-tert-butyl-1,4-benzoquinone
5-[(10Z)-14-(3,5-dihydroxyphenyl)tetradec-10-en-1-yl]benzene-1,3-diol
2-(1-{2-[(3-furylmethyl)amino]-2-oxoethyl}cyclohexyl)acetic acid
Gentisic acid
Taurochenodeoxycholic Acid (sodium salt)
jwh-018-d11
Methylmalonic acid
DL-Arginine
Glycocholic acid
-log10(FDR adjusted p) = 1.301
VIP = 1
-log10(FDR adjusted p)
VIP
CEH vs CE
Capryloylglycine
Taurolithocholic acid 3-sulfate
jwh-018-d11
Phloroglucinol
5-[(8Z,11Z)-pentadeca-8,11-dien-1-yl]benzene-1,3-diol
L-Valine
4-Aminobutyric acid
3-(3-Methoxyphenyl)propionic acid
16,16-Dimethyl prostaglandin A1
3-Oxo-7α,12α-hydroxy-5β-cholanoic acid
N-Oleoyl dopamine
L-Adrenaline
1-(4-hydroxyphenyl)propane-1,2-diol
Propionyl-L-carnitine
L-Hydroxyproline
CEM vs CE
7-Ketolithocholic acid
5-[(10Z)-14-(3,5-dihydroxyphenyl)tetradec-10-en-1-yl]benzene-1,3-diol
Glycodeoxycholic acid
Taurolithocholic acid 3-sulfate
Deoxycholic Acid
3-Oxo-7α,12α-hydroxy-5β-cholanoic acid
Taurochenodeoxycholic acid
p-Mentha-1,3,8-triene
Gentisic acid
Uridine 5'-diphosphate (UDP)
23-Nordeoxycholic acid
2,6-Di-tert-butyl-1,4-benzoquinone
N-Oleoyl dopamine
N6-Isopentenyladenosine
4-Aminobutyric acid
