# Supplementary material for: Deoxycholate induces proline provision from Escherichia coli to enhance Clostridioides difficile virulence
Source: ISME J. 2026 Sep 17;20(1):wrag249. doi: 10.1093/ismejo/wrag249 (PMC13634199; doi:10.1093/ismejo/wrag249)
Supplement: Supplementary file 1 [file supplementary_material_wrag249.zip › Figure S3.pdf]

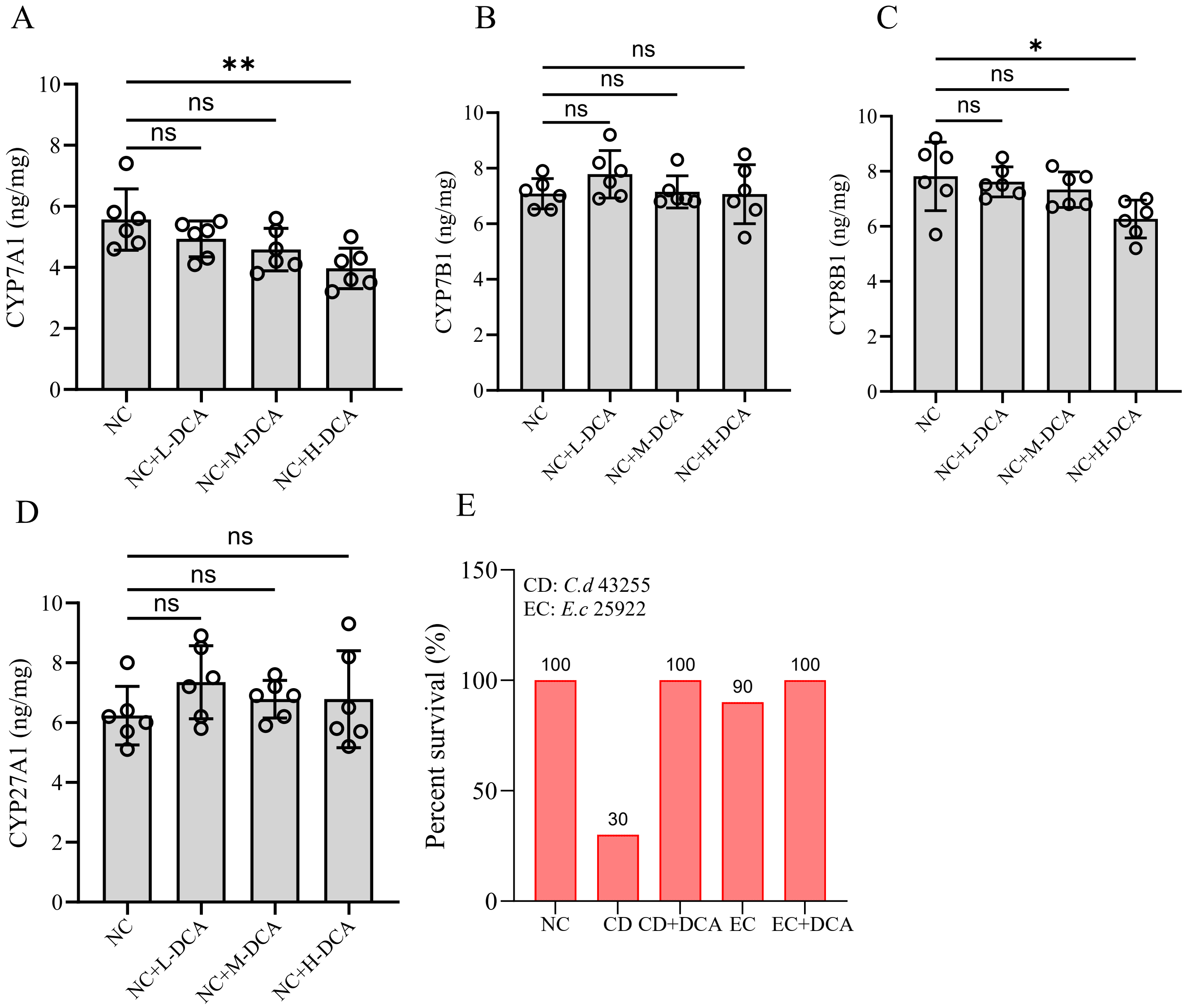

A
CYP7A1 (ng/mg)
**
ns
ns
NC
NC+L-DCA
NC+M-DCA
NC+H-DCA
B
CYP7B1 (ng/mg)
ns
ns
ns
C
CYP8B1 (ng/mg)
*
ns
ns
D
CYP27A1 (ng/mg)
ns
ns
ns
E
Percent survival (%)
CD: C.d 43255
EC: E.c 25922
100
30
100
90
100
NC
CD
CD+DCA
EC
EC+DCA
